# Structural Elucidation and Antiviral Activity Evaluation of Novelly Synthesized Guaiazulene Derivatives

**DOI:** 10.3390/md23100387

**Published:** 2025-09-28

**Authors:** Canling Cheng, Lei Hou, Xuli Tang, Guoqiang Li

**Affiliations:** 1 College of Food Science and Pharmaceutical Engineering, Zaozhuang University, Zaozhuang 277160, China; canling2010@126.com (C.C.); shiyaogongcheng@163.com (L.H.); 2 Key Laboratory of Marine Drugs, Chinese Ministry of Education, School of Medicine and Pharmacy, Ocean University of China, Qingdao 266003, China; 3 College of Chemistry and Chemical Engineering, Ocean University of China, Qingdao 266100, China; tangxvli@126.com

**Keywords:** guaiazulene derivatives, marine natural products, antiviral activities

## Abstract

A series of guaiazulene derivatives were efficiently synthesized by one-step reaction using guaiazulene as the substrate. Their structures were fully characterized by comprehensive spectroscopic methods, and their antiviral activities against influenza A (H1N1) virus were evaluated. Compounds **2b**, **2d**, **2e**, **2f**, **3a**, and **3b** exhibited significant anti-influenza activity, with IC_50_ values of 89.03 µM, 98.48 µM, 78.38 µM, 108.20 µM, 50.96 µM, and 56.09 µM, respectively. Ribavirin was used as a positive control (IC_50_ = 130.22 µM).

## 1. Introduction

Azulene, a distinctive non-benzenoid aromatic hydrocarbon, has garnered considerable interest owing to its intense blue coloration (dipole moment ≈ 1.08 D) [1,2,3,4], which arises from its fused five- and seven-membered ring structure and a unique [4+6] π-electron conjugation system [5,6]. In contrast to conventional benzenoid aromatics, azulene and its derivatives display remarkable photophysical behaviors (such as fluorescence violating Kasha’s rule), narrow optical band gaps, tunable redox properties [7,8,9,10], as well as diverse biological activities including anti-inflammatory, antibacterial, and anticancer effects [11,12,13,14,15]. These properties make azulene derivatives promising candidates for applications in organic electronics, biomedicine, supramolecular chemistry, and catalysis [16,17,18,19,20,21].

**Figure 1:**
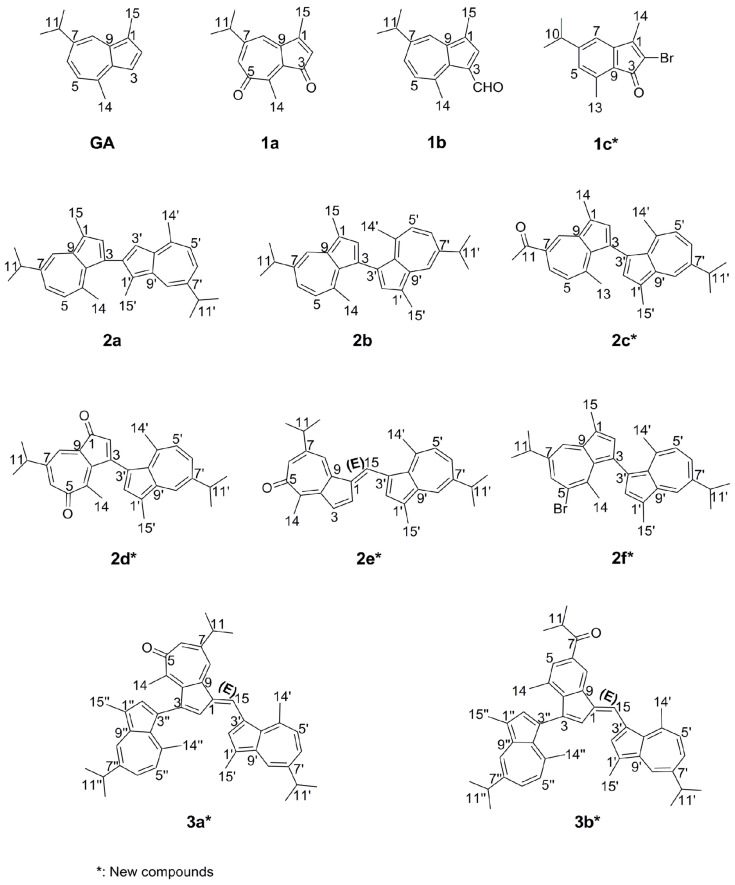
Structures of guaiazulene derivatives.

Guaiazulene (**GA**, Figure 1), chemically identified as 5-isopropyl-3,8-dimethylazulene, is a prominent derivative of azulene. It is a deep-blue sesquiterpenoid characterized by a distinctive non-benzenoid structure comprising fused seven- and five-membered rings with extensive π-conjugation. Naturally sourced from German chamomile (*Chrysanthellum indicum*) and gorgonian corals such as *Muriceides collaris* in the South China Sea, guaiazulene and related analogs like muriceidine A demonstrate notable pharmacological properties [22,23,24,25,26,27]. In recent years, growing interest has been directed toward guaiazulene due to its natural origin and favorable low toxicity profile. It has been traditionally employed in skin and mucosal repair attributable to its potent anti-inflammatory effects, and its sulfonate sodium salt serves as the key ingredient in the ulcer medication “Compound Glutamine Granules” [28]. Emerging evidence underscores its broad antiviral potential against influenza, herpesviruses, and coronaviruses, mediated through mechanisms such as viral envelope disruption, inhibition of viral protein function, and modulation of host immune responses. Although antiviral activities have been reported for various azulene and guaiazulene derivatives, their efficacy varies considerably with structural modifications, as illustrated in previous studies [29,30,31].

In our previous work, we achieved the quantitative synthesis of the natural product 7-isopropyl-1,4-dimethylazulene-3,5-dione (**1a**, Figure 1) (originally isolated from the gorgonian *Muriceides collaris* of the South China Sea and known for its strong antibacterial activity against *Vibrio anguillarum*) via an optimized one-step bromine oxidation of guaiazulene [32,33]. Reaction optimization revealed the formation of multiple byproducts with UV absorption profiles similar to that of the target compound, whose diversity was found to be condition-dependent. Acknowledging the structural and bioactive potential of azulene-type scaffolds, we adapted this synthetic approach to systematically explore the chemical space around guaiazulene, aiming to build a diversity-oriented compound library. Herein, we report the synthesis of mono-, di-, and trimeric derivatives based on guaiazulene and preliminarily investigate their structure-activity relationships through structural characterization and assessment of their anti-influenza A (H1N1) virus activities.

## 2. Results and Discussion

### 2.1. Chemistry

A series of guaiazulene derivatives were prepared through reaction of guaiazulene with Br_2_ under three distinct conditions (conditions 1–3). Eleven guaiazulene analogues were ultimately isolated using a combination of normal- and reversed-phase column chromatographic techniques, along with preparative HPLC. Among these, seven compounds (**1c**, **2c**, **2d**, **2e**, **2f**, **3a**, **3b**) were identified as previously unreported, and four (**2d**, **2e**, **3a**, **3b**) exhibited novel skeletal frameworks, as illustrated in Figure 1.

**Figure 2:**
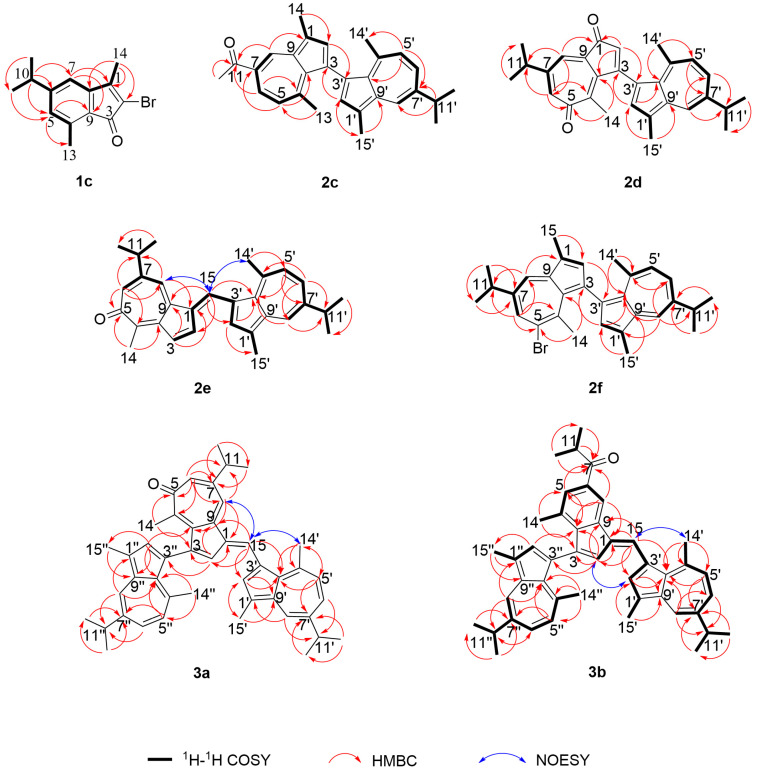
Key ^1^H-^1^H COSY, HMBC and NOESY correlations of **1c**, **2c**–**2f** and **3a**–**3b**.

Compound **1c** was obtained as yellow needles. HRESIMS analysis (Figure S1) displayed a molecular ion peak at *m*/*z* 279.0380/281.0359 [M + H]^+^, consistent with the molecular formula C_14_H_15_OBr (calcd. 279.0379/281.0359). The IR spectrum exhibited a carbonyl absorption at 1717 cm^−1^. The ^1^H NMR and ^13^C NMR (DEPT) spectra (Figures S2 and S3) revealed 14 carbon resonances (Table 1 and Table 2), comprising eight aromatic carbons, four methyl groups, one ketone carbonyl, and one aliphatic methine. All protonated carbons were unambiguously assigned using HMQC experiments (Figure 2). Based on 2D NMR analysis (Figures S4–S7), an indanone moiety was identified. An aliphatic methine proton at *δ*_H_ 2.86 (1H, m), coupled to two methyl doublets at *δ*_H_ 1.25 (6H, d, *J* = 6.9 Hz), indicated the presence of an isopropyl group. Key HMBC correlations (Figure 2) from H-10 (*δ*_H_ 2.86, m) to C-5 (*δ*_C_ 129.3) and C-7 (*δ*_C_ 116.0), along with interactions from H-5 (*δ*_H_ 6.81, s) to C-13 (*δ*_C_ 17.3), from H-7 (*δ*_H_ 6.78, s), and H-14 (*δ*_H_ 2.19, s) to C-1 (*δ*_C_ 155.6), and from H-14 to C-2 (*δ*_C_ 119.5), confirmed the attachment of the isopropyl group at C-6 (*δ*_C_ 155.0). Additionally, methyl singlets were located at C-1 and C-4 (*δ*_C_ 137.8), a carbonyl at C-3 (*δ*_C_ 190.2), and a bromine atom at C-2.

Compound **2c** was obtained as a pale green solid. HRESIMS analysis (Figure S8) established the molecular formula as C_29_H_30_O (*m*/*z* 395.2378 [M + H]^+^, calcd. 395.2369). The IR spectrum displayed a carbonyl absorption at 1733 cm^−1^. The ^1^H NMR spectrum exhibited two ABX spin systems, resonating at *δ*_H_ 8.98 (d, *J* = 1.8 Hz), 8.21 (d, *J* = 1.7 Hz), 8.08 (dd, *J* = 11.0, 1.6 Hz), 7.34 (dd, *J* = 10.6, 1.4 Hz), 6.82 (d, *J* = 10.6 Hz), and 6.81 (d, *J* = 11.0 Hz), along with two singlets at *δ*_H_ 7.53 (s), and 7.45 (s), and five methyl singlets at *δ*_H_ 2.76, 2.74, 2.69, 2.20, and 2.15 (Table 1). An aliphatic methine at *δ*_H_ 3.08 (1H, m), coupled to two methyl doublets at *δ*_H_ 1.39 (6H, d, *J* = 6.9 Hz), was consistent with the presence of an isopropyl group. Comparison of NMR (Figures S9–S15) and HRESIMS data identified **2c** as an analogue of **2b** with a distinct substituent at C-11 (*δ*_C_ 198.4) (Table 2). HMBC correlations from H-8 (*δ*_H_ 8.98, d, *J* = 1.8 Hz), H-6 (*δ*_H_ 8.08, dd, *J* = 11.0, 1.6 Hz), and H-12 (*δ*_H_ 2.74, s) to C-11 confirmed the presence of an acetyl group at this position. Additionally, correlations from H-2’ (*δ*_H_ 7.45, s) to C-3 (*δ*_C_ 126.2) and from H-2 (*δ*_H_ 7.53, s) to C-3’ (*δ*_C_ 126.2) verified the C-3/C-3’ linkage between the two guaiazulene units (Figure 2).

Compound **2d** was obtained as a dark purple solid. HRESIMS (Figure S16) established the molecular formula as C_29_H_30_O_2_ (*m*/*z* 411.2321 [M + H]^+^, calcd. 411.2319), consistent with 15 degrees of unsaturation. IR absorptions at 1735 and 1700 cm^−1^ indicated the presence of two carbonyl groups. The ^1^H NMR spectrum displayed an ABX spin system with signals at *δ*_H_ 8.22 (d, *J* = 2.1 Hz), 7.47 (dd, *J* = 10.7, 2.0 Hz), and 7.07 (d, *J* = 10.8 Hz), along with an AX spin system at *δ*_H_ 7.35 (d, *J* = 2.1 Hz), 6.86 (d, *J* = 2.1 Hz). Two singlets were observed at *δ*_H_ 7.41 (s) and 6.30 (s), in addition to three methyl singlets at *δ*_H_ 2.68, 2.65, and 1.70 (Table 1). Two aliphatic methine protons at *δ*_H_ 3.11 (1H, m) and 2.81 (1H, m) were coupled to four methyl doublets at *δ*_H_ 1.38 (6H, d, *J* = 6.9 Hz) and 1.28 (6H, d, *J* = 6.9 Hz), indicating the presence of two isopropyl groups. The combined ^1^H and ^13^C NMR (DEPT) data (Figures S17–S19) revealed 29 carbon resonances, including 18 sp^2^ carbons and two ketone carbonyl carbons. These NMR features (Figures S20–S22; Table 1 and Table 2) supported a guaiazulene dimer scaffold. Key HMBC correlations from H-2 (*δ*_H_ 6.30, s) and H-8 (*δ*_H_ 7.35, d, *J* = 2.1 Hz) to C-1 (*δ*_C_ 192.6), and from H-6 (*δ*_H_ 6.86, d, *J* = 2.1 Hz) and H-14 (*δ*_H_ 1.70, s) to C-5 (*δ*_C_ 189.0), confirmed the presence of carbonyl groups at C-1 and C-5, respectively (Figure 2). Furthermore, HMBC correlations from H-2 to C-3’ (*δ*_C_ 170.2) and from H-2’ (*δ*_H_ 7.41, s) to C-3 (*δ*_C_ 170.2) established the linkage between the two guaiazulene units via a C-C bond between C-3 and C-3’ (Figure 2).

Compound **2e** was obtained as a dark purple solid. Its molecular formula was determined to be C_30_H_32_O by HRESIMS (*m*/*z* 409.2537 [M + H]^+^, calcd. 409.2526; Figure S23), corresponding to 15 degrees of unsaturation. The IR spectrum displayed a carbonyl absorption at 1734 cm^−1^. The ^1^H NMR spectrum revealed an ABX spin system at *δ*_H_ 8.11 (d, *J* = 2.1 Hz), 7.39 (dd, *J* = 10.7, 2.0 Hz), and 7.05 (d, *J* = 10.8 Hz); an AB spin system at *δ*_H_ 7.52 (d, *J* = 5.7 Hz) and 7.27 (dd, *J* = 5.7, 1.3 Hz); an AX spin system at *δ*_H_ 7.45 (d, *J* = 0.8 Hz) and 7.14 (d, *J* = 1.5 Hz); along with two singlets at *δ*_H_ 8.22 (s) and 7.83 (s), and three methyl singlets at *δ*_H_ 3.08, 2.64, and 2.47. Two aliphatic methine protons at *δ*_H_ 3.07 (1H, m) and 2.89 (1H, m) showed coupling to four methyl doublets at *δ*_H_ 1.37 (6H, d, *J* = 6.9 Hz) and 1.31 (6H, d, *J* = 6.9 Hz), consistent with the presence of two isopropyl groups. The combined ^1^H NMR and ^13^C NMR (DEPT) data (Figures S24–S26) showed a total of 30 carbon signals, including 20 sp^2^ carbons and one ketone carbonyl carbon. These NMR characteristics (Figures S27–S30; Table 1 and Table 2) supported a guaiazulene dimer skeleton. Key HMBC correlations from H-6 (*δ*_H_ 7.14, d, *J* = 1.5 Hz) and H-14 (*δ*_H_ 2.47, s) to C-5 (*δ*_C_ 185.5) confirmed the placement of a carbonyl group at C-5 (Figure 2). Furthermore, HMBC interactions from H-15 (*δ*_H_ 8.22, s) to C-2 (*δ*_C_ 133.8), C-9 (*δ*_C_ 143.3), C-2’ (*δ*_C_ 140.0), and C-10’ (*δ*_C_ 136.6) established the linkage between the two guaiazulene units via C-1 (*δ*_C_ 136.0)/C-15 (*δ*_C_ 124.7) and C-3’ (*δ*_C_ 124.4)/C-15 bonds. The E-configuration of the Δ^1,15^ double bond was assigned based on NOESY correlations between H-15 and H-8 (*δ*_H_ 7.45, d, *J* = 0.8 Hz) and H-14’ (*δ*_H_ 3.08, s) (Figure 2).

Compound **2f** was obtained as a blue solid. Its molecular formula was assigned as C_30_H_33_Br based on ESIMS data (*m*/*z* 495.1/497.1 [M + Na]^+^ calcd. 495.1665/497.1645; Figure S31). The ^1^H NMR spectrum (Figure S32) displayed an ABX spin system at *δ*_H_ 7.97 (d, *J* = 2.1 Hz), 7.25 (dd, *J* = 10.9, 2.1 Hz) and 6.87 (d, *J* = 10.9 Hz); an AX spin system at *δ*_H_ 7.08 (d, *J* = 1.5 Hz) and 6.86 (s); along with two singlets at *δ*_H_ 6.74 (s) and 6.24 (s), as well as four methyl singlets at *δ*_H_ 3.18, 2.43, 2.14, and 1.94. Two aliphatic methine protons at *δ*_H_ 3.01 (1H, m) and 2.98 (m) were coupled to four methyl doublets at *δ*_H_ 1.34 (6H, d, *J* = 6.9 Hz) and 1.32 (6H, d, *J* = 6.9 Hz), indicating the presence of two isopropyl groups. Comparison of the NMR (Figures S33–S38) and ESIMS data (Table 1 and Table 2) revealed that **2f** is an analogue of **2b**, differing by a bromine substituent located at C-5 (*δ*_C_ 125.0). This substitution was supported by HMBC correlations from H-6 (*δ*_H_ 6.86, s) and H-14 (*δ*_H_ 1.94, s) to C-5. Additionally, HMBC interactions from H-2’ (*δ*_H_ 6.74, s) to C-3 (*δ*_C_ 139.0), and from H-2 (*δ*_H_ 6.24, s) to C-3’ (*δ*_C_ 146.1), confirmed the linkage between the two guaiazulene units via a C-C bond between C-3 and C-3’ (Figure 2).

Compound **3a** was obtained as a purple solid. Its molecular formula was determined to be C_45_H_48_O by HRESIMS (*m*/*z* 605.3783 [M + H]^+^, calcd. 605.3778; Figure S39), corresponding to 22 degrees of unsaturation. The IR spectrum showed an absorption at 1737 cm^−1^, indicating the presence of a carbonyl group. The combined ^1^H NMR and ^13^C NMR (DEPT) data (Figures S40–S42) revealed a total of 45 carbon signals, including 30 sp^2^-hybridized carbons, 11 methyl groups, and one ketone carbonyl carbon. Analysis of the HMBC data indicated that the remaining 10 sp^2^ carbons, along with a conjugated ketone (*δ*_C_ 186.9, C-5), belonged to a transformed guaiazulenyl unit structurally analogous to that in compound **2e**. These NMR characteristics (Figures S43–S45; Table 3) supported the assignment of **3a** as a guaiazulene trimer. The connectivity among the three guaiazulene units was established by key HMBC correlations: from H-15 (*δ*_H_ 8.22, s) to C-2 (*δ*_C_ 137.7), C-2’ (*δ*_C_ 139.0), C-9 (*δ*_C_ 144.5), and C-10’ (*δ*_C_ 132.9); from H-2 (*δ*_H_ 7.38, d, *J* = 1.2 Hz) to C-3” (*δ*_C_ 124.8); and from H-2” (*δ*_H_ 7.82, s) to C-3 (*δ*_C_ 125.3). These interactions confirmed linkages via C-1 (*δ*_C_ 134.5)/C-15 (*δ*_C_ 122.3), C-3’ (*δ*_C_ 148.1)/C-15, and C-3/C-3” bonds. Additionally, the E-configuration of the Δ^1,15^ double bond was assigned based on the NOESY correlation between H-15 and both H-8 (*δ*_H_ 7.54, s) and H-14’ (*δ*_H_ 2.65, s) (Figure 2).

Compound **3b** was obtained as a bright red solid with the molecular formula C_45_H_48_O. HRESIMS (Figure S46) showed a molecular ion peak at *m*/*z* 604.3710 [M]^+^ (calcd. 604.3700), and the IR spectrum indicated the presence of a carbonyl group (1738 cm^−1^). Comparison of NMR spectroscopic data revealed that **3b** shares the same partial structure as **3a**, comprising two guaiazulene units. The NMR features (Figures S47–S49; Table 3) were consistent with a guaiazulene trimer. Based on 2D NMR analysis (Figures S50–S53), an indanone moiety was identified. An isobutanoyl group was assigned based on ^1^H-^1^H COSY correlations between a methine proton (*δ*_H_ 3.68) and two equivalent methyl protons (overlapped at *δ*_H_ 1.27), along with HMBC correlations from the methyl protons to a ketone carbon (*δ*_C_ 204.9, C-7). The attachment of the isobutanoyl group at C-6 (*δ*_C_ 145.8) and a methyl group at C-4 (*δ*_C_ 131.5) was deduced from HMBC correlations from H-5 (*δ*_H_ 7.58, s) and H-8 (*δ*_H_ 8.30, s) to C-7, and from H-14 (*δ*_H_ 1.85, s) to C-5 (*δ*_C_ 129.5) and C-10 (*δ*_C_ 144.7). HMBC correlations from H-15 (*δ*_H_ 8.49, s) to C-2 (*δ*_C_ 130.7), C-2’ (*δ*_C_ 141.0), C-9 (*δ*_C_ 137.9), and C-10’ (*δ*_C_ 136.5), together with correlations from H-2 (*δ*_H_ 7.20, s) to C-3” (*δ*_C_ 131.0) and from H-2” (*δ*_H_ 7.55, s) to C-3 (*δ*_C_ 124.4), confirmed the connection of one indanone unit to two guaiazulene subunits via C-1 (*δ*_C_ 133.7)/C-15 (*δ*_C_ 127.0), C-3’ (*δ*_C_ 125.1)/C-15, and C-3/C-3” bonds. Additionally, the E-configuration of the Δ^1, 15^ double bond was assigned based on NOESY correlations between H-15 and H-14’ (*δ*_H_ 3.19, s), as well as between H-2 and H-2’ (*δ*_H_ 7.94, s) (Figure 2).

### 2.2. Antiviral Activity

In our previous studies, guaiazulene derivatives have demonstrated promising potential as anti-influenza A virus (IAV) agents [30]. As a continuation of this research, we further evaluated the antiviral activities of guaiazulene-derived compounds (**GA**, **1a**–**1c**, **2a**–**2f**, **3a**–**3b**) against the replication of influenza A H1N1 virus in MDCK cells (Table 4). Among these, compounds **2b**, **2d**, **2e**, **2f**, **3a**, and **3b** exhibited significant antiviral effects, with IC_50_ values of 89.03, 98.48, 78.38, 108.20, 50.96, and 56.09 μM, respectively, using ribavirin (IC_50_ = 130.22 μM) as a positive control.

Compared to the positive control ribavirin, compounds **GA**, **2b**, **2d**, **2e**, **3a**, and **3b** exhibited potent inhibitory activity against influenza A (H1N1) virus, while compounds **2a**, **2c**, and **2f** showed only weak inhibition. In contrast, compounds **1a**, **1b**, and **1c** demonstrated no detectable inhibitory activity. Notably, based on structural classification, trimers and dimers generally possessed higher antiviral activity than monomers.

Guaiazulene exhibited potent anti-H1N1 activity, whereas its monomeric derivatives **1a**, **1b**, and **1c** showed no inhibitory effects. This suggests that oxidation or bromination of the guaiazulene core or side chains abolishes antiviral efficacy in monomeric compounds. Among the dimeric derivatives, compound **2b** demonstrated strong antiviral activity, while **2a**, **2c**, and **2f** exhibited only weak inhibition. These results indicate that a 3, 3’-symmetric linkage between azulene units enhances antiviral potency, whereas a 2, 3’-asymmetric connection reduces activity. Furthermore, structural modifications such as oxidation or bromination on the core or side chains appear to compromise antiviral function.

## 3. Materials and Methods

### 3.1. The General Procedures

All chemicals were used as received without further purification. Guaiazulene (purity > 98%), used as the starting material, was obtained from Beijing Hengye Zhongyuan Chemical Co., Ltd (Beijing, China). NMR spectra were recorded on a JEOL JNM-ECP 600 or a Bruker Avance-500 FT NMR spectrometer using tetramethylsilane (TMS) as an internal standard, and chemical shifts are reported in δ values. IR spectra were acquired on a Nicolet Nexus 470 spectrophotometer using KBr pellets. UV spectra were measured with a Beckman DU 640 spectrophotometer. ESI-MS analyses were performed on a Q-TOF Ultima Global GAA076 LC mass spectrometer. Semipreparative reversed-phase HPLC was carried out on a Waters 2695 system (with a 2998 detector) using ODS columns [YMC-Pack ODS-A (10 μm, 10 mm × 250 mm, flow rate: 1.5 mL/min) and Kromasil (10 μm, 10 mm × 250 mm, flow rate: 1.5 mL/min)]. Thin-layer chromatography (TLC) was conducted on silica gel GF254 plates, and column chromatography (CC) was performed using silica gel (300–400 mesh) from Qingdao Marine Chemical Factory.

### 3.2. Synthesis of Guaiazulene Derivatives (Conditions 1–3)

Building upon references [34,35,36,37,38,39,40,41,42,43] and optimized synthetic protocols established in preliminary studies, we designed and carried out the reaction of guaiazulene with bromine under three distinct conditions-varying solvent polarity (protic vs. aprotic), acid/base catalysis, and temperature. This strategy afforded a series of guaiazulene derivatives featuring structurally diverse frameworks.

**Figure 3:**
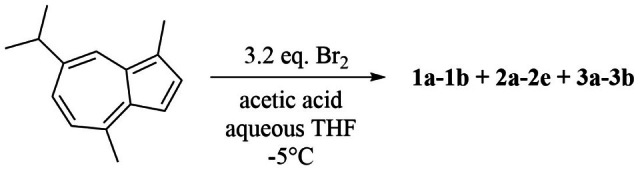
The synthesis of guaiazulene derivatives **1a**–**1b**, **2a**–**2e** and **3a**–**3b** under condition 1.

Condition 1: A stirred solution of guaiazulene (**GA**, 1.20 g, 6.05 mmol) in 80% aqueous THF (230 mL) was maintained at −5 °C. Acetic acid (1.40 mL) was added, followed by dropwise addition of a solution of bromine (1.20 mL) in THF (10 mL) (Figure 3). The reaction progress was monitored by TLC and quenched with ethyl acetate (1:1, *v*/*v*). The mixture was first washed with saturated aqueous NaHSO_3_ solution (1:1, *v*/*v*) to remove excess bromine, HBr, and acetic acid. Subsequently, it was washed with water (1:1, *v*/*v*) to eliminate salts, and the aqueous phase was back-extracted with ethyl acetate (1:1, *v*/*v*). Each washing and extraction step was repeated three times. The combined organic phases were concentrated under reduced pressure at 40 °C using a rotary evaporator to afford the crude product. The residue was purified by silica gel column chromatography with a stepwise gradient of petroleum ether/ethyl acetate (from 100:1 to 10:1, *v*/*v*), followed by HPLC, yielding nine compounds: **1a** (43.2 mg, 3.6%; ODS, 10 µm, 10 mm × 250 mm; MeOH/H_2_O, 90:10, *v*/*v*; 1.5 mL/min; *t*_R_ = 9.5 min), **1b** (4.8 mg, 0.4%; ODS, 10 µm, 10 mm × 250 mm; MeOH/H_2_O, 90:10, *v*/*v*; 1.5 mL/min; *t*_R_ = 13.5 min), **2a** (16.8 mg, 1.4%; ODS, 10 µm, 10 mm × 250 mm; MeCN/H_2_O, 90:10, *v*/*v*; 1.5 mL/min; *t*_R_ = 15.0 min), **2b** (55.2 mg, 4.6%; ODS, 10 µm, 10 mm × 250 mm; MeCN/H_2_O, 90:10, *v*/*v*; 1.5 mL/min; *t*_R_ = 15.4 min), **2c** (4.8 mg, 0.4%; ODS, 10 µm, 10 mm × 250 mm; MeOH/H_2_O, 90:10, *v*/*v*; 1.5 mL/min; *t*_R_ = 18.1 min), **2d** (3.6 mg, 0.3%; ODS, 10 µm, 10 mm × 250 mm; MeOH/H_2_O, 90:10, *v*/*v*; 1.5 mL/min; *t*_R_ = 16.3 min), **2e** (24.0 mg, 2.0%; ODS, 10 µm, 10 mm × 250 mm; MeOH/H_2_O, 90:10, *v*/*v*; 1.5 mL/min; *t*_R_ = 36.3 min), **3a** (393.6 mg, 32.8%; ODS, 10 µm, 10 mm × 250 mm; MeCN/H_2_O, 90:10, *v*/*v*; 1.5 mL/min; *t*_R_ = 25.0 min), **3b** (4.8 mg, 0.4%; ODS, 10 µm, 10 mm × 250 mm; MeCN/H_2_O, 90:10, *v*/*v*; 1.5 mL/min; *t*_R_ = 33.2 min).

As shown in Figure 1 and Figure 3, the reaction under this condition primarily yielded a series of oxidized products, with no brominated compounds detected. This outcome is likely due to the high reactivity of the protic solvent system combined with acid catalysis, which promoted rapid hydrolysis and oxidation of any brominated intermediates immediately after their formation. Consequently, we adjusted the reaction conditions by employing an aprotic solvent and basic catalysis to explore whether the desired brominated compounds could be synthesized.

**Figure 4:**
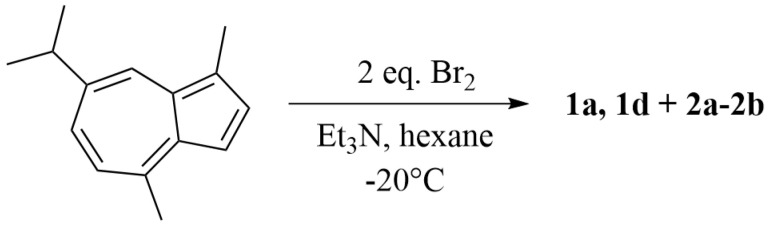
The synthesis of guaiazulene derivatives **1a**, **1d** and **2a**–**2b** under condition 2.

Condition 2: A solution of guaiazulene (**GA**, 0.12 g, 0.60 mmol) in hexane (10 mL) was maintained at −20 °C. Triethylamine (0.10 mL) was added, followed by dropwise addition of bromine (0.08 mL) in hexane (2 mL) (Figure 4). The reaction was monitored by TLC and quenched with water (1:1, *v*/*v*). The mixture was washed with water (1:1, *v*/*v*) to remove salts, and the aqueous phase was back-extracted with ethyl acetate (1:1, *v*/*v*). Each extraction step was repeated three times. The combined ethyl acetate phases were concentrated under reduced pressure at 40 °C using a rotary evaporator to afford the crude product. Purification by silica gel column chromatography with a stepwise gradient of petroleum ether/ethyl acetate (from 100:1 to 10:1, *v*/*v*), followed by HPLC, afforded three compounds: **1a** (1.2 mg, 1.0%; ODS, 10 µm, 10 mm × 250 mm; MeOH/H_2_O, 90:10, *v*/*v*; 1.5 mL/min; *t*_R_ = 9.5 min), **2a** (1.68 mg, 1.4%; ODS, 10 µm, 10 mm × 250 mm; MeCN/H_2_O, 90:10, *v*/*v*; 1.5 mL/min; *t*_R_ = 15.0 min), **2b** (2.76 mg, 2.3%; ODS, 10 µm, 10 mm × 250 mm; MeCN/H_2_O, 90:10, *v*/*v*; 1.5 mL/min; *t*_R_ = 15.4 min).

As shown in Figure 1 and Figure 4, the reactivity under this condition was relatively low. Although side-chain brominated compounds were obtained, the product structural diversity remained limited. Nevertheless, the coexistence of both brominated and oxidized products suggests that brominated intermediates exhibit considerable stability under basic conditions. Therefore, based on the outcomes of the first two conditions, we further modified the reaction system by employing a protic solvent under basic catalysis to explore whether a series of brominated-oxidized products could be obtained.

**Figure 5:**
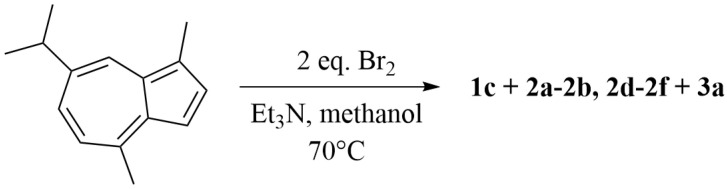
The synthesis of guaiazulene derivatives **1c, 2a**–2**b**, **2d**–**2f** and **3a** under condition 3.

Condition 3: A solution of guaiazulene (**GA**, 0.36 g, 1.80 mmol) in methanol (30 mL) was maintained at 70 °C. Triethylamine (0.30 mL) was added, followed by dropwise addition of bromine (0.24 mL) in THF (6 mL) (Figure 5). The reaction was monitored by TLC and quenched with ethyl acetate (1:1, *v*/*v*). After cooling to room temperature, the mixture was concentrated under reduced pressure at 40 °C using a rotary evaporator to afford the crude product. The residue was dissolved in ethyl acetate (1:1, *v*/*v*) and washed with water (1:1, *v*/*v*) to remove salts. The aqueous phase was back-extracted with ethyl acetate (1:1, *v*/*v*), and each extraction step was repeated three times. The combined organic phases were concentrated under reduced pressure at 40 °C. Purification by silica gel column chromatography using a stepwise gradient of petroleum ether/ethyl acetate (from 100:1 to 10:1, *v*/*v*), followed by HPLC, yielded seven compounds: **1c** (2.9 mg, 0.8%; ODS, 10 µm, 10 mm × 250 mm; MeOH/H_2_O, 90:10, *v*/*v*; 1.5 mL/min; *t*_R_ = 18.5 min), **2a** (33.1 mg, 9.2%; ODS, 10 µm, 10 mm × 250 mm; MeCN/H_2_O, 90:10, *v*/*v*; 1.5 mL/min; *t*_R_ = 15.0 min), **2b** (110.5 mg, 30.7%; ODS, 10 µm, 10 mm × 250 mm; MeCN/H_2_O, 90:10, *v*/*v*; 1.5 mL/min; *t*_R_ = 15.4 min), **2d** (8.3 mg, 2.3%; ODS, 10 µm, 10 mm × 250 mm; MeOH/H_2_O, 90:10, *v*/*v*; 1.5 mL/min; *t*_R_ = 16.3 min), **2e** (11.5 mg, 3.2%; ODS, 10 µm, 10 mm × 250 mm; MeOH/H_2_O, 90:10, *v*/*v*; 1.5 mL/min; *t*_R_ = 36.3 min), **2f** (3.2 mg, 0.9%; ODS, 10 µm, 10 mm × 250 mm; MeCN/H_2_O, 90:10, *v*/*v*; 1.5 mL/min; *t*_R_ = 16.1 min), **3a** (182.2 mg, 50.6%; ODS, 10 µm, 10 mm × 250 mm; MeCN/H_2_O, 90:10, *v*/*v*; 1.5 mL/min; *t*_R_ = 25.0 min).

As shown in Figure 1 and Figure 5, the products obtained under this condition exhibited greater diversity compared to those from the previous two conditions. Furthermore, the bromination site shifted from the side chain to the parent nucleus, affording a mono-brominated guaiazulene dimer (**2f**) and a mono-brominated indenone monomer (**1c**).

As demonstrated above, the described reactions have successfully achieved structural diversification of guaiazulene derivatives. Analysis under the three specified conditions revealed the following: 1. Guaiazulene dimers were consistently formed across all conditions, indicating a high propensity for polymerization within this conjugated system. The polymers primarily featured 3,3’-symmetric linkages, underscoring the enhanced reactivity at the C-3 position. Brominated and oxidized products were predominantly observed at the C-3, C-5, and C-1 sites, further corroborating the relative reactivity order in azulene systems: C-3 > C-5 > C-1. 2. Under protic solvent conditions, the system demonstrated high reactivity, affording structurally diverse and complex products. Acid catalysis predominantly led to oxidation products, whereas base catalysis yielded both brominated and oxidized compounds, indicating that brominated intermediates remain relatively stable under alkaline media. Minor products formed through intramolecular carbon rearrangement and decarbonization pathways consistent with previously documented mechanisms. Notably, this study is the first to report novel polymeric frameworks (compounds **2e**, **3a**, **3b**) containing unprecedented double-bond linkages.

7-Isopropyl-1,4-dimethylazulene-3,5-dione (**1a**): yellow needles; ESI-MS 229.1 [M+H]^+^; ^1^H NMR (600 MHz, CDCl_3_): δ 6.74 (1H, d, *J* = 1.6 Hz), 6.62 (1H, d, *J* = 1.3 Hz), 6.22 (1H, s), 2.78–2.71 (1H, m), 2.62 (3H, s), 2.28 (3H, d, *J* = 1.1 Hz), 1.24 (6H, d, *J* = 6.8 Hz). The present findings broadly align with literature reports [37].

7-Isopropyl-1,4-dimethylazulene-3-carbaldehyde (**1b**): dark red solid; EI-MS 226.1 [M]^+^; ^1^H NMR (CDCl_3_, 600 MHz) δ 10.64 (1H, s), 8.29 (1H, d, *J* = 2.0 Hz), 8.23 (1H, s), 7.59 (1H, dd, *J* = 10.8, 2.0 Hz), 7.43 (1H, d, *J* = 10.8 Hz), 3.18–3.14 (1H, m), 3.15 (3H, s), 2.59 (3H, s), 1.39 (6H, d, *J* = 6.9 Hz). The present findings broadly align with literature reports [37].

2-Bromo-6-isopropyl-1,4-dimethyl-3H-inden-3-one (**1c**): yellow oil; UV (CH_2_Cl_2_) max (log ε) 254 (2.77), 238 (2.80) nm; IR (KBr) νmax 1717, 1637, 1601, 1548, 1463, 1372, 1069 cm^−1^; ^1^H NMR and ^13^C NMR spectroscopic data are summarized in Table 1 and Table 2, respectively; HRESIMS *m*/*z* 279.0380/281.0359 [M+H]^+^ (calcd for C_14_H_16_BrO 279.0379/281.0359).

7,7-Diisopropyl-1,1,4,4-tetramethyl-2’,3-biazulene (**2a**): yellow green solid; ESI-MS 394.1 [M]+; ^1^H NMR (CDCl_3_, 600 MHz): δ 8.23 (1H, d, *J* = 2.0 Hz), 8.21 (1H, d, *J* = 1.8 Hz), 7.59 (1H, s), 7.39 (1H, dd, *J* = 10.6, 1.8 Hz), 7.38 (1H, dd, *J* = 10.6, 1.8 Hz), 7.27 (1H, s), 7.07 (1H, d, *J* = 10.6 Hz), 6.91 (1H, d, *J* = 10.7 Hz), 3.16–3.12 (1H, m), 3.11–3.07 (1H, m), 2.83 (3H, s), 2.72 (3H, s), 2.50 (3H, s), 2.37 (3H, s), 1.41 (6H, d, *J* = 6.9 Hz), 1.40 (6H, d, *J* = 6.9 Hz). The present findings broadly align with literature reports [37].

7,7-Diisopropyl-1,1,4,4-tetramethyl-3,3-biazulene (**2b**): dark green solid; ESI-MS 394.1 [M]^+^; ^1^H NMR (CDCl_3_, 600 MHz) δ 8.20 (1H, d, *J* = 2.0 Hz), 7.47 (1H, s), 7.31 (1H, dd, J = 10.6, 2.0 Hz), 6.79 (1H, d, J = 10.6 Hz), 3.11–3.04 (1H, m), 2.70 (3H, s), 2.20 (3H, s), 1.39 (6H, dd, J = 6.9 Hz). The present findings broadly align with literature reports [37].

1-(7-Isopropyl-1,1,4,4-tetramethyl-3,3-biazulen-7-yl)ethanone (**2c**): pale green solid; UV (CH_2_Cl_2_) max (log ε) 315 (3.07), 293 (3.09), 249 (3.09) nm; IR (KBr) νmax 1733, 1687, 1635, 1543, 1460, 1432, 1369, 1158 cm^−1^; ^1^H NMR and ^13^C NMR spectroscopic data are summarized in Table 1 and Table 2, respectively; HRESIMS *m*/*z* 395.2378 [M+H]^+^ (calcd for C_29_H_31_O 395.2369).

7,7-Diisopropyl-1,4,4-trimethyl-3,3-biazulene-1,5-dione (**2d**): dark purple solid; UV (CH_2_Cl_2_) max (log ε) 355 (3.38), 296 (3.84), 252 (3.97) nm; IR (KBr) νmax 1735, 1700, 1688, 1631, 1543, 1460, 1457, 1430, 1370, 1166 cm^−1^; ^1^H NMR and ^13^C NMR spectroscopic data are summarized in Table 1 and Table 2, respectively; HRESIMS *m*/*z* 411.2321 [M+H]^+^ (calcd for C_29_H_31_O_2_ 411.2319).

(E)-7-Isopropyl-1-((7-isopropyl-1,4-dimethylazulen-3-yl)methylene)-4-methylazulen-5(1H)-one (**2e**): dark purple solid; UV (CH_2_Cl_2_) max (log ε) 326 (2.75), 283 (2.99), 238 (3.04), 228 (3.04) nm; IR (KBr) νmax 1734, 1682, 1630, 1546, 1461, 1434, 1376, 1160 cm^−1^; ^1^H NMR and ^13^C NMR spectroscopic data are summarized in Table 1 and Table 2, respectively; HRESIMS *m*/*z* 409.2537 [M+H]^+^ (calcd for C_30_H_33_O 409.2526).

(E)-5-Bromo-7,7-diisopropyl-1,1,4,4-tetramethyl-3,3-biazulen (**2f**): blue solid; UV (CH_2_Cl_2_) max (log ε) 377 (1.94), 360 (2.06), 312 (2.63), 291 (2.74), 252 (2.68), 232 (2.69) nm; IR (KBr) νmax 1651, 1596, 1548, 1455, 1437, 1379, 1165 cm^−1^; ^1^H NMR and ^13^C NMR spectroscopic data are summarized in Table 1 and Table 2, respectively; ESIMS *m*/*z* 495.1/497.1 [M+Na]^+^.

(E)-7,7-Diisopropyl-1-((7-isopropyl-1,4-dimethylazulen-3-yl)methylene)-1,4,4-trimethyl-3,3-biazulen-5(1H)-one (**3a**): purple solid; UV (CH_2_Cl_2_) max (log ε) 294 (3.15), 243 (3.14) nm; IR (KBr) νmax 1737, 1696, 1632, 1544, 1450, 1432, 1371, 1156 cm^−1^; ^1^H NMR and ^13^C NMR spectroscopic data are summarized in Table 3; HRESIMS *m*/*z* 605.3783 [M+H]^+^ (calcd for C_45_H_49_O 605.3778).

(E)-1-(3-(7-Isopropyl-1,4-dimethylazulen-3-yl)-1-((7-isopropyl-1,4-dimethylazulen-3-yl)methylene)-4-methyl-1H-inden-6-yl)-2-methylpropan-1-one (**3b**): bright red solid; UV (CH_2_Cl_2_) max (log ε) 292 (3.24), 255 (3.35), 238 (3.38) nm; IR (KBr) νmax 1738, 1686, 1593, 1547, 1463, 1372, 1161 cm^−1^; ^1^H NMR and ^13^C NMR spectroscopic data are summarized in Table 3; HRESIMS *m*/*z* 604.3710 [M]^+^ (calcd for C_45_H_48_O 604.3700).

### 3.3. Anti-H1N1 Activity Assay

The antiviral activities of the guaiazulene derivatives were evaluated against influenza A H1N1 virus using the cytopathic effect (CPE) inhibition assay and the MTT method. In the MTT assay, cell lines were cultured in RPMI-1640 medium supplemented with 10% fetal bovine serum (FBS) at 37 °C under a humidified atmosphere containing 5% CO_2_ and 95% air. Cell suspensions (200 μL) at a density of 5 × 10^4^ cells/mL were seeded into 96-well microtiter plates and incubated for 24 h. Subsequently, 2 μL of each test solution (in methanol) was added to the wells, and the plates were further incubated for 72 h. After this period, 20 μL of MTT solution (5 mg/mL in RPMI-1640 medium) was added to each well, and the plates were incubated for an additional 4 h. The medium containing MTT was then carefully removed, and dimethyl sulfoxide (DMSO) was added to dissolve the formazan crystals. The absorbance was measured at 540 nm using a Spectra Max Plus microplate reader. Dose–response curves were plotted, and the IC_50_ values, defined as the concentration of compound required to inhibit 50% of cell proliferation, were calculated from the linear regression of the log-dose response curves.

The antiviral activity against H1N1 virus was assessed using a cytopathic effect (CPE) inhibition assay [44]. Confluent MDCK cell monolayers were incubated with influenza virus (A/Puerto Rico/8/34 (H1N1), PR/8) at 37 °C for 1 h. After removing the viral inoculum, the cells were maintained in infection medium (RPMI 1640 supplemented with 4 μg/mL trypsin) containing various concentrations of the test compounds at 37 °C. Following 48 h of incubation, the cells were fixed with 100 μL of 4% formaldehyde for 20 min at room temperature. After removing the formaldehyde, the cells were stained with 0.1% crystal violet for 30 min. The plates were then washed, dried, and the intensity of crystal violet staining in each well was measured at 570 nm using a microplate reader (Bio-Rad Laboratories, Inc., Hercules, CA, USA). The IC_50_ value was defined as the concentration of compound required to inhibit 50% of CPE production at 48 h post-infection.

## 4. Conclusions

Guaiazulene derivatives serve as a structural motif of interest that connects traditional natural products with modern antiviral research. Their distinctive non-benzenoid aromatic architecture and potential multi-target mechanisms offer a valuable source of chemical diversity for developing agents against viral infections. In this study, eleven guaiazulene derivatives were synthesized from guaiazulene through various polymerization strategies, yielding monomers, dimers, and trimers. Among these, seven new compounds were identified, four of which possess previously unreported core skeletons. This diversity-oriented synthesis successfully expanded the structural repertoire of guaiazulene derivatives, suggesting new directions for molecular template design. Additionally, several compounds exhibited promising anti-influenza virus activity, providing initial insights into structure–activity relationships (SARs) and potential lead structures for future antiviral development. These findings support further investigation into the antiviral properties and structural optimization of this class of compounds.

## Figures and Tables

**Table 1:** ^1^H NMR data of guaiazulene derivatives **1c**, **2c**–**2f** (CDCl_3_).

No.	1c	2c	2d	2e	2f
	*δ*_H_ ^a^ (*J* in Hz)	*δ*_H_ ^a^ (*J* in Hz)	*δ*_H_ ^b^ (*J* in Hz)	*δ*_H_ ^b^ (*J* in Hz)	*δ*_H_ ^b^ (*J* in Hz)
1					
2		7.53, s	6.30, s	7.27, dd (5.7, 1.3)	6.24, s
3				7.52, d (5.7)	
4					
5	6.81, s	6.81, d (11.0)			
6		8.08, dd (11.0, 1.6)	6.86, d (2.1)	7.14, d (1.5)	6.86, s
7	6.78, s				
8		8.98, d (1.8)	7.35, d (2.1)	7.45, d (0.8)	7.08, d (1.5)
9					
10	2.86, m				
11	1.25, d (6.9)		2.81, m	2.89, m	2.98, m
12	1.25, d (6.9)	2.74, s	1.28, d (6.9)	1.31, d (6.9)	1.32, d (6.9)
13	2.49, s	2.20, s	1.28, d (6.9)	1.31, d (6.9)	1.32, d (6.9)
14	2.19, s	2.76, s	1.70, s	2.47, s	1.94, s
15				8.22, s	2.14, s
1’					
2’		7.45, s	7.41, s	7.83, s	6.74, s
3’					
4’					
5’		6.82, d (10.6)	7.07, d (10.8)	7.05, d (10.8)	6.87, d (10.9)
6’		7.34, dd (10.6, 1.4)	7.47, dd (10.7, 2.0)	7.39, dd (10.7, 2.0)	7.25, dd (10.9, 1.8)
7’					
8’		8.21, d (1.7)	8.22, d (2.1)	8.11, d (2.1)	7.97, d (2.1)
9’					
10’					
11’		3.08, m	3.11, m	3.07, m	3.01, m
12’		1.39, d (6.9)	1.38, d (6.9)	1.37, d (6.9)	1.34, d (6.9)
13’		1.39, d (6.9)	1.38, d (6.9)	1.37, d (6.9)	1.34, d (6.9)
14’		2.15, s	2.68, s	3.08, s	3.18, s
15’		2.69, s	2.65, s	2.64, s	2.43, s

^a^ Spectra recorded at 500 MHz. ^b^ Spectra recorded at 600 MHz.

**Table 2:** ^13^C NMR data of guaiazulene derivatives **1c**, **2c**–**2f** (CDCl_3_) ^c^.

No.	1c	2c	2d	2e	2f
	*δ*_C_ ^d^	*δ*_C_ ^d^	*δ*_C_ ^e^	*δ*_C_ ^e^	*δ*_C_ ^e^
1	155.6, C	135.8, C	192.6, C	136.0, C	146.1, C
2	119.5, C	141.9, CH	134.4 ^g^, CH	133.8, CH	137.0, CH
3	190.2, C	126.2, C	170.2, C	135.2, CH	139.0, C
4	137.8, C	152.3, C	145.0, C	140.7, C	146.1, C
5	129.3, CH	125.6, CH	189.0, C	185.5, C	125.0, C
6	155.0, C	136.4, CH	136.1, CH	135.1, CH	126.0, CH
7	116.0, CH	127.2, C	154.3, C	152.4, C	147.9, C
8	145.6, C	133.7, CH	127.9, CH	122.4, CH	114.7, CH
9	124.3, C	134.8, C	137.3, C	143.3, C	124.5, C
10	34.5, CH	133.0, C	143.4, C	146.9, C	133.1, C
11	23.5, CH_3_	198.4, C	37.2, CH	38.5, CH	34.2, CH
12	23.5, CH_3_	26.7, CH_3_	22.3, CH_3_	23.3, CH_3_	24.4, CH_3_
13	17.3, CH_3_	26.6, CH_3_	22.3, CH_3_	23.3, CH_3_	24.4, CH_3_
14	13.0, CH_3_	13.2, CH_3_	17.4, CH_3_	17.5, CH_3_	19.0, CH_3_
15				124.7, CH	13.1, CH_3_
1’		138.1, C	125.9, C	127.0, C	133.1, C
2’		140.4, CH	136.4, CH	140.0, CH	136.9, CH
3’		126.2, C	122.2, C	124.4, C	146.1, C
4’		146.3, C	146.0, C	147.0, C	144.1, C
5’		126.5, CH	128.5, CH	129.4, CH	125.0, CH
6’		135.0, CH	136.1, CH	136.2, CH	133.9, CH
7’		139.7, C	142.2, C	143.1, C	139.2, C
8’		134.0, CH	134.5 ^g^, CH	134.5, CH	132.8, CH
9’		124.1, C	139.4, C	141.1, C	125.0, C
10’		132.2, C	139.4, C	136.6, C	131.9, C
11’		37.9, CH	38.0, CH	37.9, CH	37.6, CH
12’		24.7 ^f^, CH_3_	24.6, CH_3_	24.4, CH_3_	24.6 ^h^, CH_3_
13’		24.6 ^f^, CH_3_	24.6, CH_3_	24.4, CH_3_	24.5 ^h^, CH_3_
14’		26.5, CH_3_	27.1, CH_3_	28.5, CH_3_	29.7, CH_3_
15’		12.9, CH_3_	12.9, CH_3_	13.0, CH_3_	12.9, CH_3_

^c^ The assignments were based on HMQC and HMBC spectra. ^d^ Spectra recorded at 125 MHz. ^e^ Spectra recorded at 150 MHz. ^f,g,h^ Both carbons can be interchanged.

**Table 3:** ^1^H NMR and ^13^C NMR data of guaiazulene derivatives **3a**–**3b** (CDCl_3_) ^c^.

No.	3a		3b	
	*δ*_H_ ^b^ (*J* in Hz)	*δ*_C_ ^e^	*δ*_H_ ^a^ (*J* in Hz)	*δ*_C_ ^d^
1		134.5, C		133.7, C
2	7.38, d (1.2)	137.7, CH	7.20, s	130.7, CH
3		125.3, C		124.4, C
4		144.1, C		131.5, C
5		186.9, C		129.5, CH
6	7.12, s	134.0 ^i^, CH		145.8, C
7		152.1, C		204.9, C
8	7.54, s	121.7, CH	8.30, s	116.4, CH
9		144.5, C		137.9, C
10		146.5, C		144.7, C
11	2.94, m	38.1, CH	3.68, m	35.2, CH
12	1.37, d (6.9)	23.3 ^j^, CH_3_	1.27, d (6.9)	19.6 ^l^, CH_3_
13	1.37, d (6.9)	23.2 ^j^, CH_3_	1.27, d (6.9)	19.5 ^l^, CH_3_
14	1.87, s	18.2, CH_3_	1.85, s	19.1, CH_3_
15	8.22, s	122.3, CH	8.49, s	127.0, CH
1’		124.7 ^k^, C		132.5, C
2’	7.46, s	139.0, CH	7.94, s	141.0, CH
3’		148.1, C		125.1, C
4’		146.2, C		147.1, C
5’	6.92, d (10.8)	126.9, CH	7.06, d (10.9)	129.4, CH
6’	7.36, dd (10.8, 1.8)	135.2, CH	7.37, dd (10.9, 1.8)	136.0, CH
7’		140.1, C		143.1, C
8’	8.20, d (2.0)	134.0 ^i^, CH	8.06, d (1.8)	134.2, CH
9’		138.0, C		140.9, C
10’		132.9, C		136.5, C
11’	3.08, m	37.8, CH	3.05, m	37.9, CH
12’	1.39, d (6.9)	24.4, CH_3_	1.35, d (6.9)	24.4, CH_3_
13’	1.39, d (6.9)	24.4, CH_3_	1.35, d (6.9)	24.4, CH_3_
14’	2.65, s	26.7, CH_3_	3.19, s	28.9, CH_3_
15’	2.68, s	12.9, CH_3_	2.56, s	12.9, CH_3_
1”		127.0, C		124.4 ^m^, C
2”	7.82, s	139.8, CH	7.55, s	139.4, CH
3”		124.8 ^k^, C		131.0 ^m^, C
4”		146.8, C		146.5, C
5”	7.03, d (10.8)	129.2, CH	6.91, d (10.7)	126.7, CH
6”	7.35, dd (10.8, 1.8)	136.0, CH	7.35, dd (10.7, 1.8)	135.1, CH
7”		143.0, C		139.7, C
8”	8.05, d (1.9)	134.3, CH	8.20, d (1.8)	133.8, CH
9”		140.7, C		138.5, C
10”		136.3, C		133.7, C
11”	3.04, m	37.9, CH	3.08, m	37.9, CH
12”	1.35, d (6.9)	24.6, CH_3_	1.38, d (6.9)	24.7 ^n^, CH_3_
13”	1.35, d (6.9)	24.6, CH_3_	1.38, d (6.9)	24.6 ^n^, CH_3_
14”	3.14, s	28.6, CH_3_	2.61, s	26.3, CH_3_
15”	2.55, s	12.9, CH_3_	2.69, s	12.9, CH_3_

^a^ Spectra recorded at 500 MHz. ^b^ Spectra recorded at 600 MHz. ^c^ The assignments were based on HMQC and HMBC spectra. ^d^ Spectra recorded at 125 MHz. ^e^ Spectra recorded at 150 MHz. ^i–n^ Both carbons can be interchanged.

**Table 4:** Antiviral activities of guaiazulene derivatives on replication of H1N1 influenza A virus in MDCK cells.

Compounds	Concentration (μg/mL)	Inhibition (%)	IC_50_ (μg/mL)	IC_50_ (μM)
Ribavirin	50	72.9	31.8	130.22
GA	50	70.2	36.8	185.73
1a	50	4.5	>100	>438.37
1b	50	9.6	>100	>442.20
1c	50	0.0	>100	>358.37
2a	50	32.7	81.3	206.2
2b	50	75.1	35.1	89.03
2c	50	41.9	67.5	171.22
2d	50	67.9	40.4	98.48
2e	50	63.8	32.0	78.38
2f	50	43.4	51.2	108.20
3a	50	67.5	30.8	50.96
3b	50	69.5	33.9	56.09

## Data Availability

The data for the research results can be obtained from Supporting Information.

## Supplementary Materials

The following supporting information can be downloaded at https://www.mdpi.com/article/10.3390/md23100387/s1, Figure S1. The HR-ESI-MS spectrum of **1c**; Figure S2. The ^1^H-NMR spectrum of **1c** in CDCl_3_; Figure S3. The APT spectrum of **1c** in CDCl_3_; Figure S4. The HMQC spectrum of **1c** in CDCl_3_; Figure S5. The HMBC spectrum of **1c** in CDCl_3_; Figure S6. The ^1^H-^1^H COSY spectrum of **1c** in CDCl_3_; Figure S7. The NOESY spectrum of **1c** in CDCl_3_; Figure S8. The HR-ESI-MS spectrum of **2c**; Figure S9. The ^1^H-NMR spectrum of **2c** in CDCl_3_; Figure S10. The ^13^C-NMR spectrum of **2c** in CDCl_3_; Figure S11. The DEPT spectrum of **2c** in CDCl_3_; Figure S12. The HMQC spectrum of **2c** in CDCl_3_; Figure S13. The HMBC spectrum of **2c** in CDCl_3_; Figure S14. The ^1^H-^1^H COSY spectrum of 2c in CDCl_3_; Figure S15. The NOESY spectrum of **2c** in CDCl_3_; Figure S16. The HR-ESI-MS spectrum of **2d**; Figure S17. The ^1^H-NMR spectrum of **2d** in CDCl_3_; Figure S18. The ^13^C-NMR spectrum of **2d** in CDCl_3_; Figure S19. The DEPT spectrum of **2d** in CDCl_3_; Figure S20. The HMBC spectrum of **2d** in CDCl_3_; Figure S21. The ^1^H-^1^H COSY spectrum of **2d** in CDCl_3_; Figure S22. The NOESY spectrum of **2d** in CDCl_3_; Figure S23. The HR-ESI-MS spectrum of **2e**; Figure S24. The ^1^H-NMR spectrum of **2e** in CDCl_3_; Figure S25. The ^13^C-NMR spectrum of **2e** in CDCl_3_; Figure S26. The DEPT spectrum of **2e** in CDCl_3_; Figure S27. The HMQC spectrum of **2e** in CDCl_3_; Figure S28. The HMBC spectrum of **2e** in CDCl_3_; Figure S29. The ^1^H-^1^H COSY spectrum of **2e** in CDCl_3_; Figure S30. The NOESY spectrum of **2e** in CDCl_3_; Figure S31. The HR-ESI-MS spectrum of **2f**; Figure S32. The 1H-NMR spectrum of **2f** in CDCl_3_; Figure S33. The ^13^C-NMR spectrum of **2f** in CDCl_3_; Figure S34. The DEPT spectrum of **2f** in CDCl_3_; Figure S35. The HMQC spectrum of **2f** in CDCl_3_; Figure S36. The HMBC spectrum of **2f** in CDCl_3_; Figure S37. The ^1^H-^1^H COSY spectrum of **2f** in CDCl_3;_ Figure S38. The NOESY spectrum of **2f** in CDCl_3_; Figure S39. The HR-ESI-MS spectrum of **3a**; Figure S40. The ^1^H-NMR spectrum of **3a** in CDCl_3_; Figure S41. The ^13^C-NMR spectrum of **3a** in CDCl_3_; Figure S42. The DEPT spectrum of **3a** in CDCl_3_; Figure S43. The HMQC spectrum of **3a** in CDCl_3_; Figure S44. The HMBC spectrum of **3a** in CDCl_3_; Figure S45. The NOESY spectrum of **3a** in CDCl_3_; Figure S46. The HR-ESI-MS spectrum of **3b**; Figure S47. The ^1^H-NMR spectrum of **3b** in CDCl_3_; Figure S48. The ^13^C-NMR spectrum of **3b** in CDCl_3_; Figure S49. The DEPT spectrum of **3b** in CDCl_3_; Figure S50. The HMQC spectrum of **3b** in CDCl_3_; Figure S51. The HMBC spectrum of **3b** in CDCl_3_; Figure S52. The ^1^H-^1^H COSY spectrum of **3b** in CDCl_3_; Figure S53. The NOESY spectrum of **3b** in CDCl_3_.

## Abbreviations

HRESIMS	High-resolution electrospray ionization mass spectrometry
NMR	Nuclear magnetic resonance
DEPT	Distortionless enhancement by polarization transfer
HMBC	^1^H-detected heteronuclear multiple bond connectivity
HMQC	^1^H-detected heteronuclear multiple quantum coherence
COSY	Shift correlation spectroscopy
NOESY	Nuclear Overhauser effect spectroscopy
^1^H-NMR	^1^H-nuclear magnetic resonance
^13^C-NMR	^13^C-nuclear magnetic resonance
IR	Infrared absorption spectrum
UV	Ultraviolet-visible spectrum
EI MS	Electron impact ionization mass spectra
ESI MS	Electrospray ionization mass spectra
dd	Doublet of doublet
*m*/*z*	Mass to charge ratio
ppm	Part per million
MTT	3-(4, 5-dimethyl-2-thiazoyl)-2, 5-diphenyl-tetrazolium bromide
SRB	Sulforhodamine B
HPLC	High performance liquid chromatography
TLC	Thin-layer chromatography
MDCK	Madin-Darby canine kidney
CPE	Cytopathic effect
SAR	Structure-activity relationship
