# Supplementary material for: Structural Elucidation and Antiviral Activity Evaluation of Novelly Synthesized Guaiazulene Derivatives
Source: Mar Drugs. 2025 Sep 28;23(10):387. doi: 10.3390/md23100387 (PMC12565720; doi:10.3390/md23100387)
Supplement: Supplementary file 1 [file marinedrugs-23-00387-s001.zip › 0821╓o│┼▓─┴╧.pdf]

SUPPORTING INFORMATION

Structural elucidation and antiviral activity evaluation of novelly synthesized guaiazulene derivatives

Canling Cheng [1,2], Lei Hou [1], Xuli Tang [3] and Guoqiang Li [2,*]

1 College of Food Science and Pharmaceutical Engineering, Zaozhuang University, Zaozhuang 277160, People's Republic of China; canling2010@126.com (C.C.); shiyaogongcheng@163.com (L.H.)

2 Key Laboratory of Marine Drugs, Chinese Ministry of Education, School of Medicine and Pharmacy, Ocean University of China, Qingdao 266003, People's Republic of China

3 College of Chemistry and Chemical Engineering, Ocean University of China, Qingdao 266100, People's Republic of China; tangxvli@126.com

** Correspondence: liguoqiang@ouc.edu.cn (G.L.); Tel.: +86-532-8203-2323 (G.L.)*

List of Supporting Information

Figure S1. The HR-ESI-MS spectrum of **1c**……………………………………………1

Figure S2. The ^{1}H-NMR spectrum of **1c** in $CDCl_3$ (500 MHz)……………………1

Figure S3. The APT spectrum of **1c** in $CDCl_3$ (125 MHz)……………………………2

Figure S4. The HMQC spectrum of **1c** in $CDCl_3$ (500 MHz)…………………………2

Figure S5. The HMBC spectrum of **1c** in $CDCl_3$ (500 MHz)…………………………3

Figure S6. The ^{1}H-^{1}H COSY spectrum of **1c** in $CDCl_3$ (500 MHz)………………3

Figure S7. The NOESY spectrum of **1c** in $CDCl_3$ (500 MHz)…………………………4

Figure S8. The HR-ESI-MS spectrum of **2c**……………………………………………4

Figure S9. The ^{1}H-NMR spectrum of **2c** in $CDCl_3$ (500 MHz)……………………5

Figure S10. The ^{13}C-NMR spectrum of **2c** in $CDCl_3$ (125 MHz)…………………5

Figure S11. The DEPT spectrum of **2c** in $CDCl_3$ (125 MHz)…………………………6

Figure S12. The HMQC spectrum of **2c** in $CDCl_3$ (500 MHz)…………………………6

Figure S13. The HMBC spectrum of **2c** in $CDCl_3$ (500 MHz)…………………………7

Figure S14. The ^{1}H-^{1}H COSY spectrum of **2c** in $CDCl_3$ (500 MHz)………………7

Figure S15. The NOESY spectrum of **2c** in $CDCl_3$ (500 MHz)…………………………8

Figure S16. The HR-ESI-MS spectrum of **2d**……………………………………………8

Figure S17. The ^{1}H-NMR spectrum of **2d** in $CDCl_3$ (600 MHz)……………………9

Figure S18. The ^{13}C-NMR spectrum of **2d** in $CDCl_3$ (150 MHz)…………………9

Figure S19. The DEPT spectrum of **2d** in $CDCl_3$ (150 MHz)…………………………10

Figure S20. The HMBC spectrum of **2d** in $CDCl_3$ (600 MHz)…………………………10

Figure S21. The ^{1}H-^{1}H COSY spectrum of **2d** in $CDCl_3$ (600 MHz)………………11

Figure S22. The NOESY spectrum of **2d** in $CDCl_3$ (600 MHz)…………………………11

Figure S23. The HR-ESI-MS spectrum of **2e**……………………………………………12

Figure S24. The ^{1}H-NMR spectrum of **2e** in $CDCl_3$ (600 MHz)……………………12

Figure S25. The ^{13}C-NMR spectrum of **2e** in $CDCl_3$ (150 MHz)…………………13

Figure S26. The DEPT spectrum of **2e** in $CDCl_3$ (150 MHz)…………………………13

Figure S27. The HMQC spectrum of **2e** in $CDCl_3$ (600 MHz)…………………………14

Figure S28. The HMBC spectrum of **2e** in $CDCl_3$ (600 MHz)…………………………14

Figure S29. The ^{1}H-^{1}H COSY spectrum of **2e** in $CDCl_3$ (600 MHz)....................................15

Figure S30. The NOESY spectrum of **2e** in $CDCl_3$ (600 MHz)...15

Figure S31. The HR-ESI-MS spectrum of **2f**..16

Figure S32. The ^{1}H-NMR spectrum of **2f** in $CDCl_3$ (600 MHz)...................................16

Figure S33. The ^{13}C-NMR spectrum of **2f** in $CDCl_3$...17

Figure S34. The DEPT spectrum of **2f** in $CDCl_3$..17

Figure S35. The HMQC spectrum of **2f** in $CDCl_3$ (600 MHz)...18

Figure S36. The HMBC spectrum of **2f** in $CDCl_3$ (600 MHz)..18

Figure S37. The ^{1}H-^{1}H COSY spectrum of **2f** in $CDCl_3$ (600 MHz)...................................19

Figure S38. The NOESY spectrum of **2f** in $CDCl_3$ (600 MHz)..19

Figure S39. The HR-ESI-MS spectrum of **3a**..20

Figure S40. The ^{1}H-NMR spectrum of **3a** in $CDCl_3$ (600 MHz)...................................20

Figure S41. The ^{13}C-NMR spectrum of **3a** in $CDCl_3$ (150 MHz).................................21

Figure S42. The DEPT spectrum of **3a** in $CDCl_3$ (150 MHz)..21

Figure S43. The HMQC spectrum of **3a** in $CDCl_3$ (600 MHz)..22

Figure S44. The HMBC spectrum of **3a** in $CDCl_3$ (600 MHz)...22

Figure S45. The NOESY spectrum of **3a** in $CDCl_3$ (600 MHz)...23

Figure S46. The HR-ESI-MS spectrum of **3b**..23

Figure S47. The ^{1}H-NMR spectrum of **3b** in $CDCl_3$ (500 MHz)...................................24

Figure S48. The ^{13}C-NMR spectrum of **3b** in $CDCl_3$ (125 MHz).................................24

Figure S49. The DEPT spectrum of **3b** in $CDCl_3$ (125 MHz)..25

Figure S50. The HMQC spectrum of **3b** in $CDCl_3$ (500 MHz)..25

Figure S51. The HMBC spectrum of **3b** in $CDCl_3$ (500 MHz)...26

Figure S52. The ^{1}H-^{1}H COSY spectrum of **3b** in $CDCl_3$ (500 MHz)...................................26

Figure S53. The NOESY spectrum of **3b** in $CDCl_3$ (500 MHz)...27

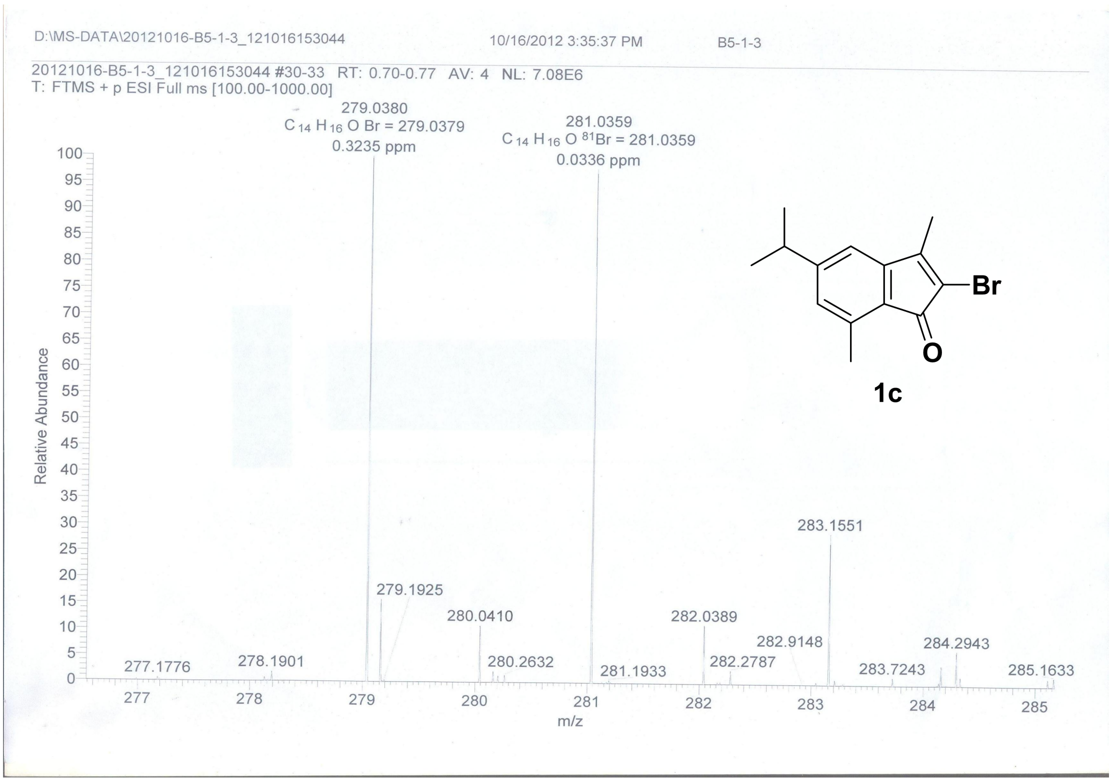

Figure S1. The HR-ESI-MS spectrum of **1c**.

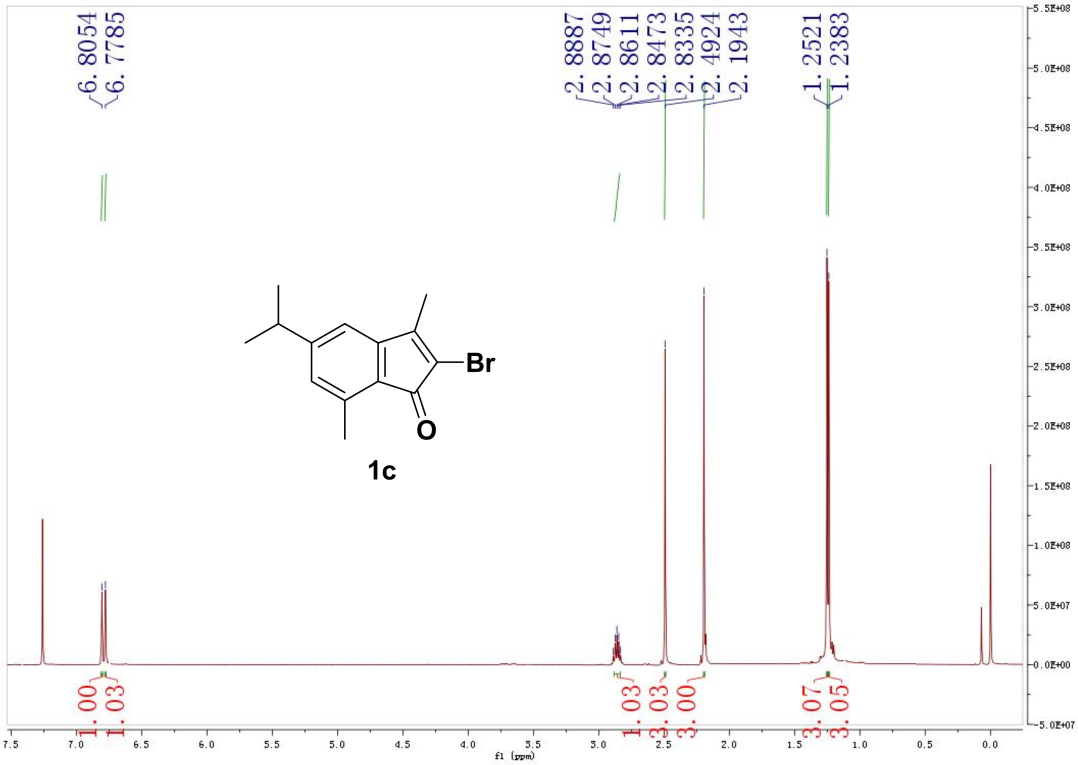

Figure S2. The ^{1}H-NMR spectrum of **1c** in $CDCl_3$ (500 MHz).

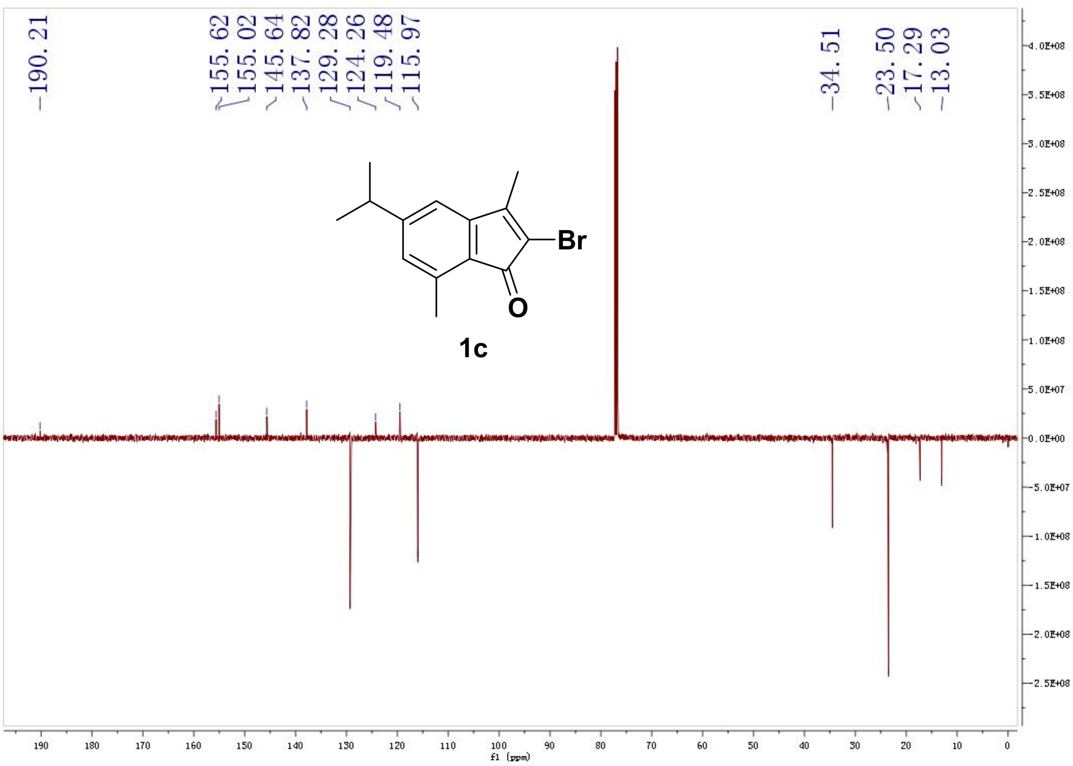

Figure S3. The APT spectrum of **1c** in $CDCl_3$ (125 MHz).

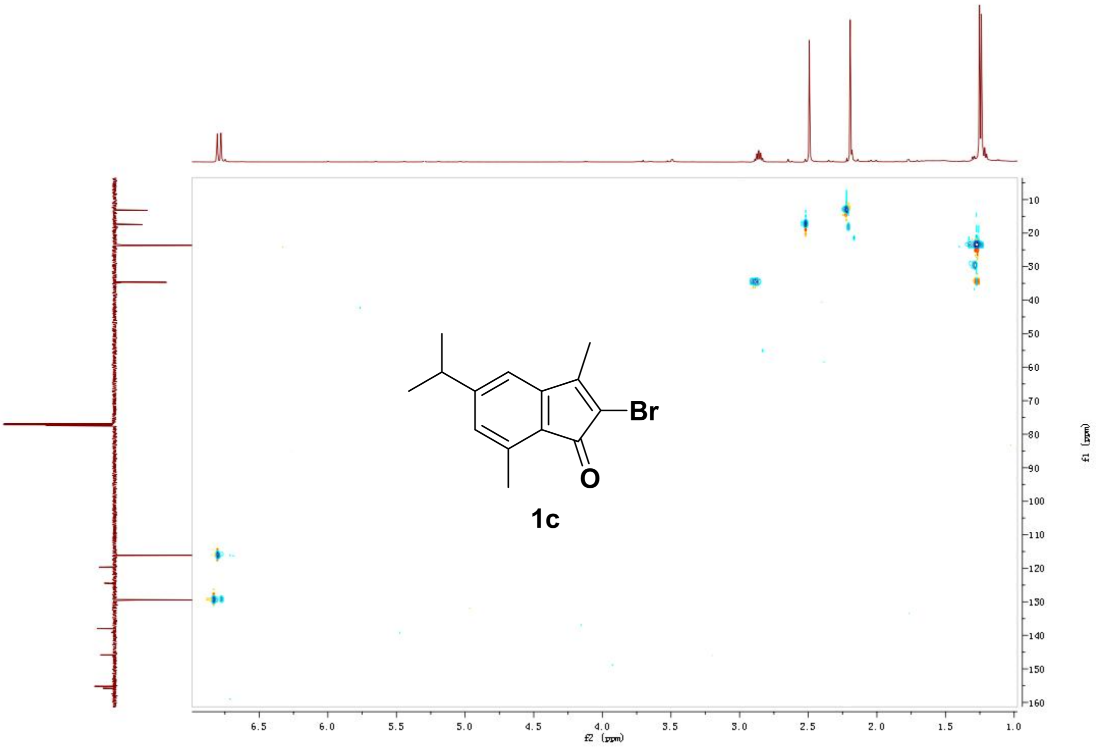

Figure S4. The HMQC spectrum of **1c** in $CDCl_3$ (500 MHz).

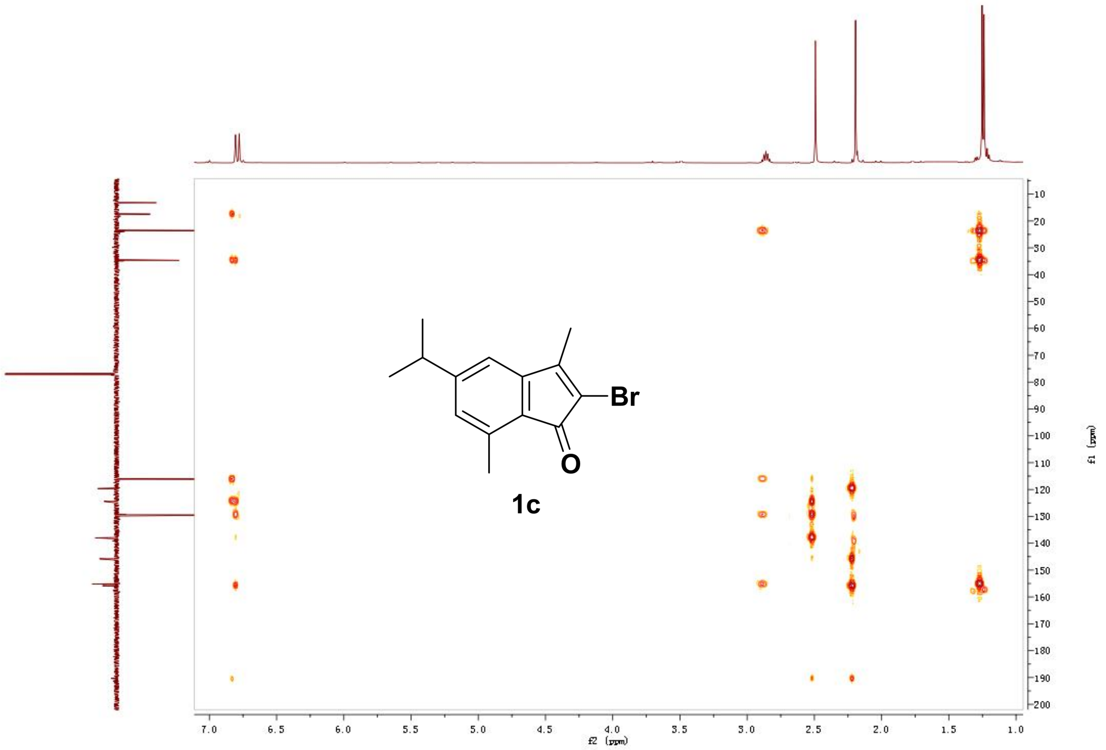

Figure S5. The HMBC spectrum of **1c** in $CDCl_3$ (500 MHz).

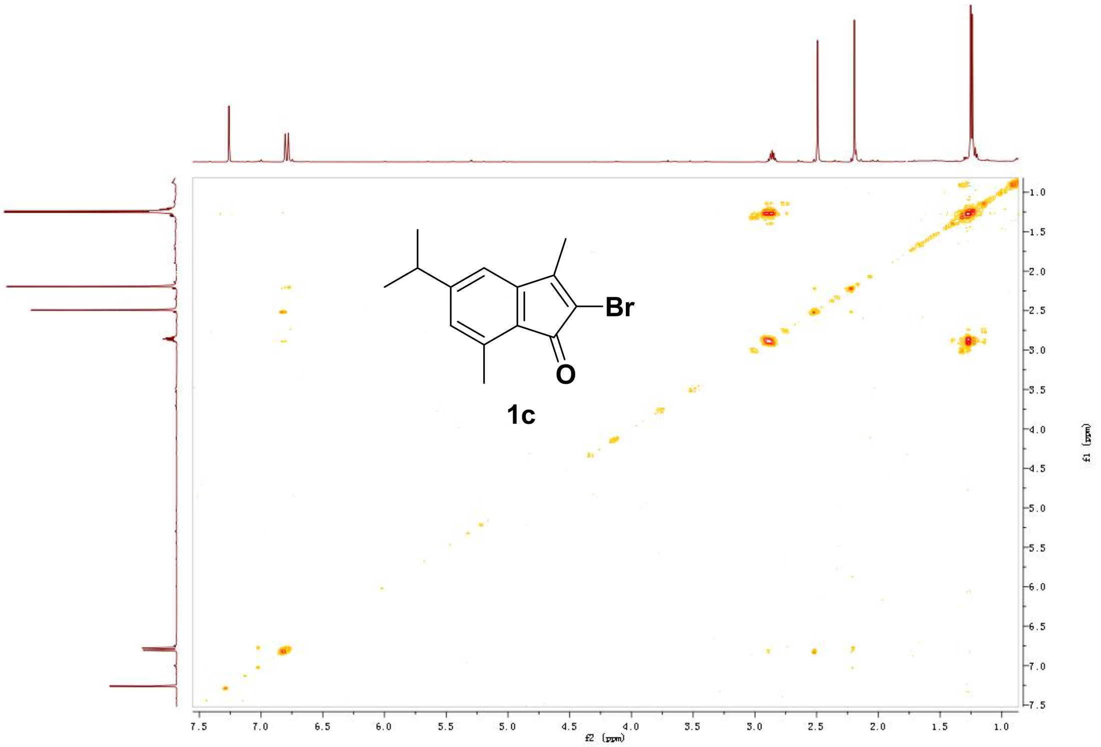

Figure S6. The ^1H-^1H COSY spectrum of **1c** in $CDCl_3$ (500 MHz).

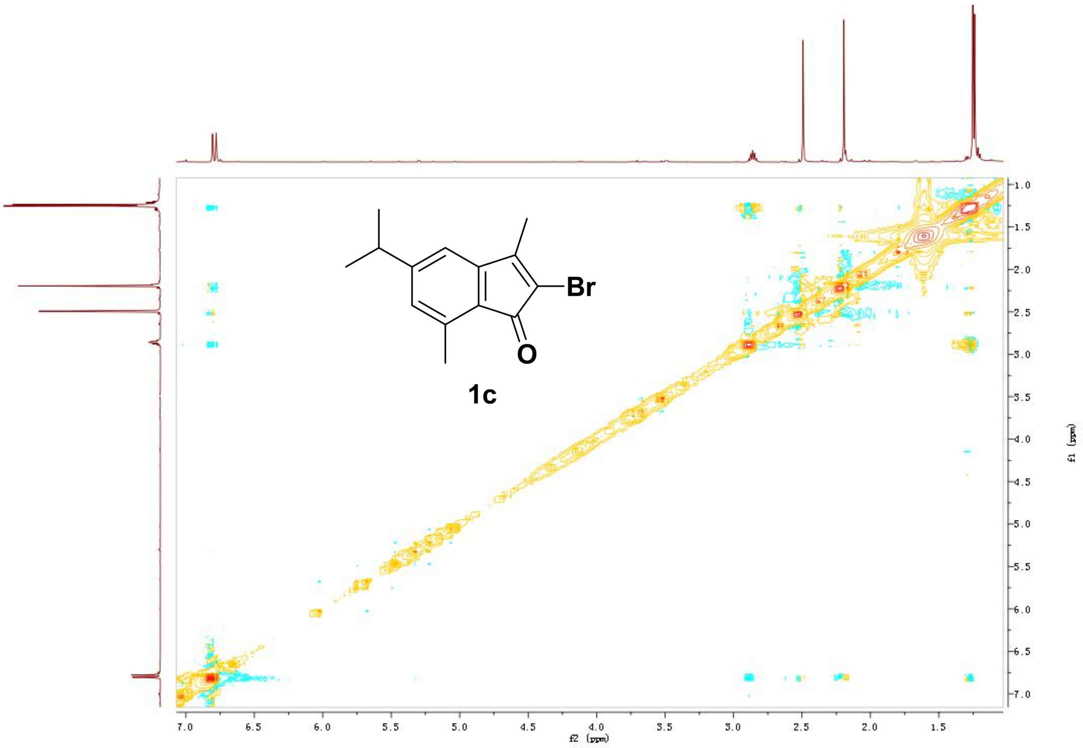

Figure S7. The NOESY spectrum of **1c** in $CDCl_3$ (500 MHz).

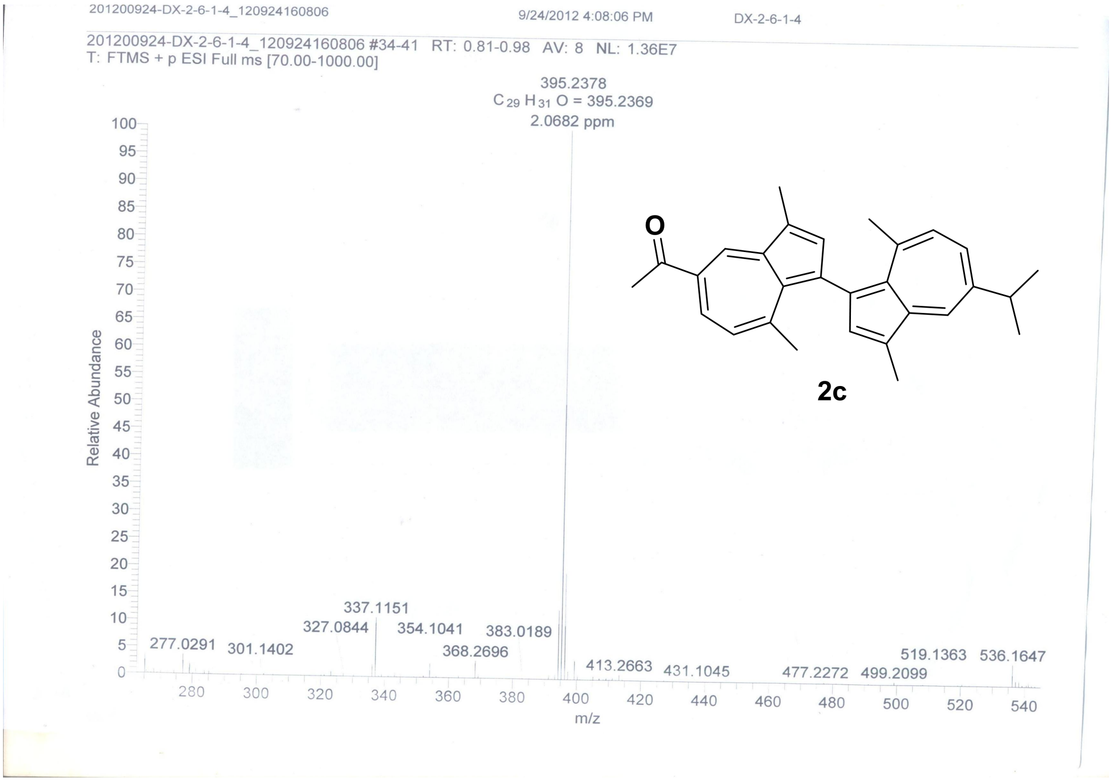

Figure S8. The HR-ESI-MS spectrum of **2c**.

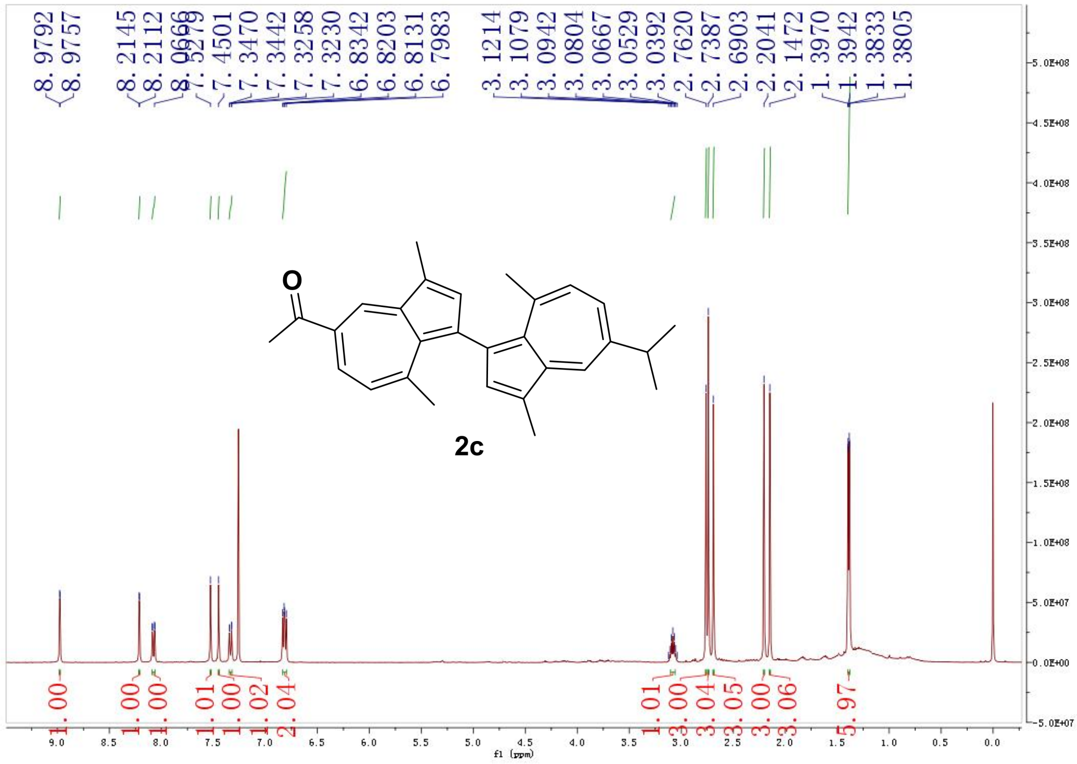

Figure S9. The ^{1}H-NMR spectrum of **2c** in $CDCl_3$ (500 MHz).

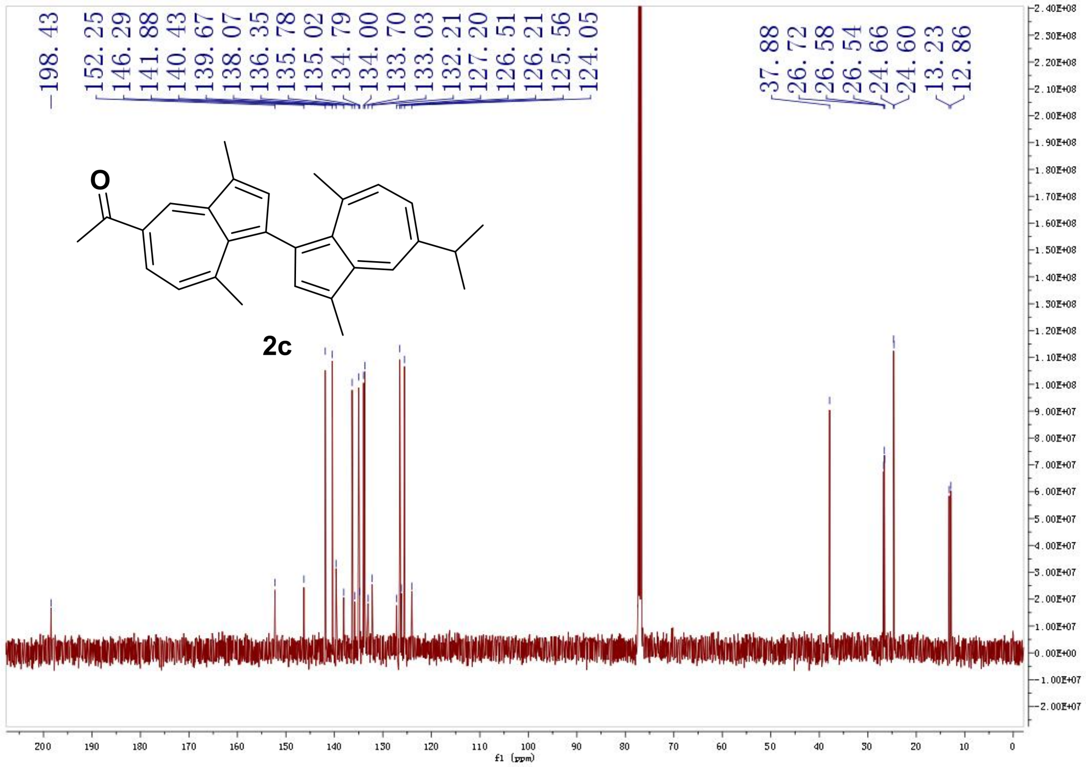

Figure S10. The ^{13}C-NMR spectrum of **2c** in $CDCl_3$ (125 MHz).

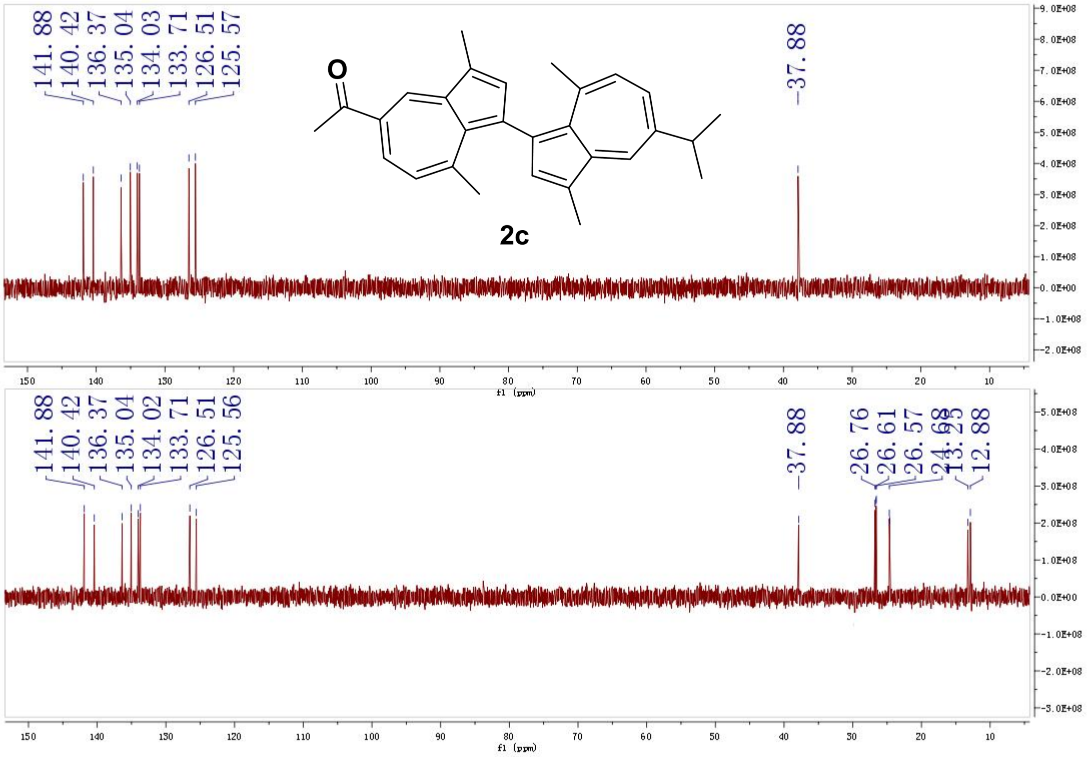

Figure S11. The DEPT spectrum of **2c** in $CDCl_3$ (125 MHz).

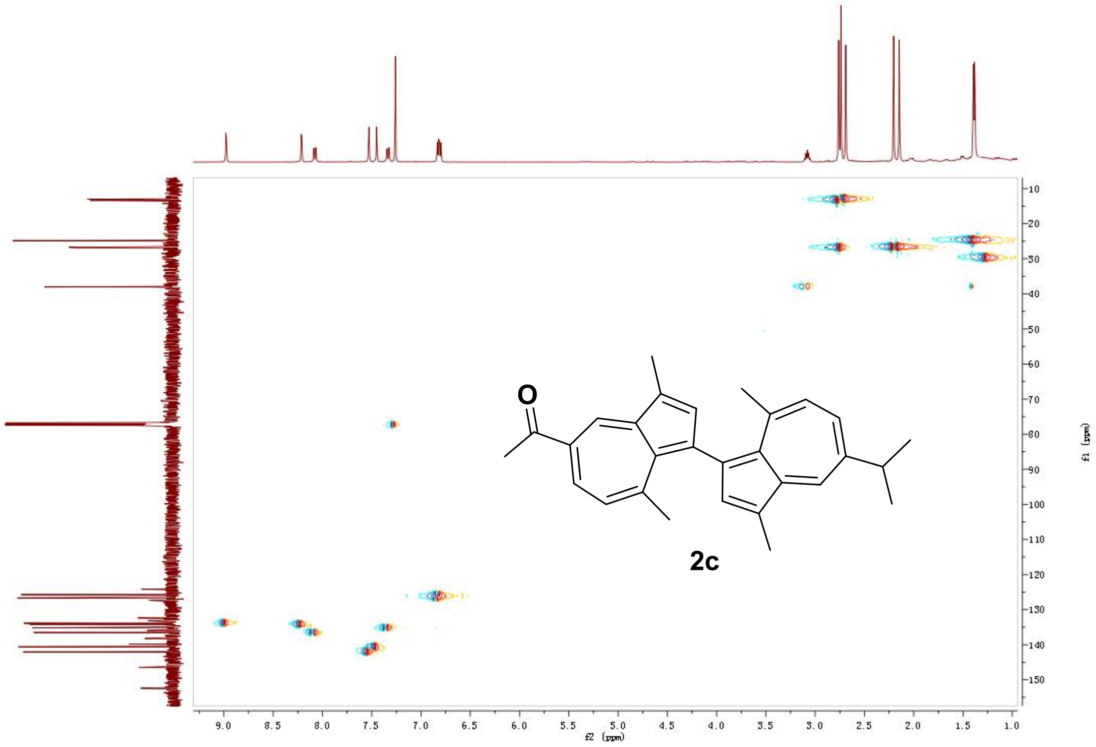

Figure S12. The HMQC spectrum of **2c** in $CDCl_3$ (500 MHz).

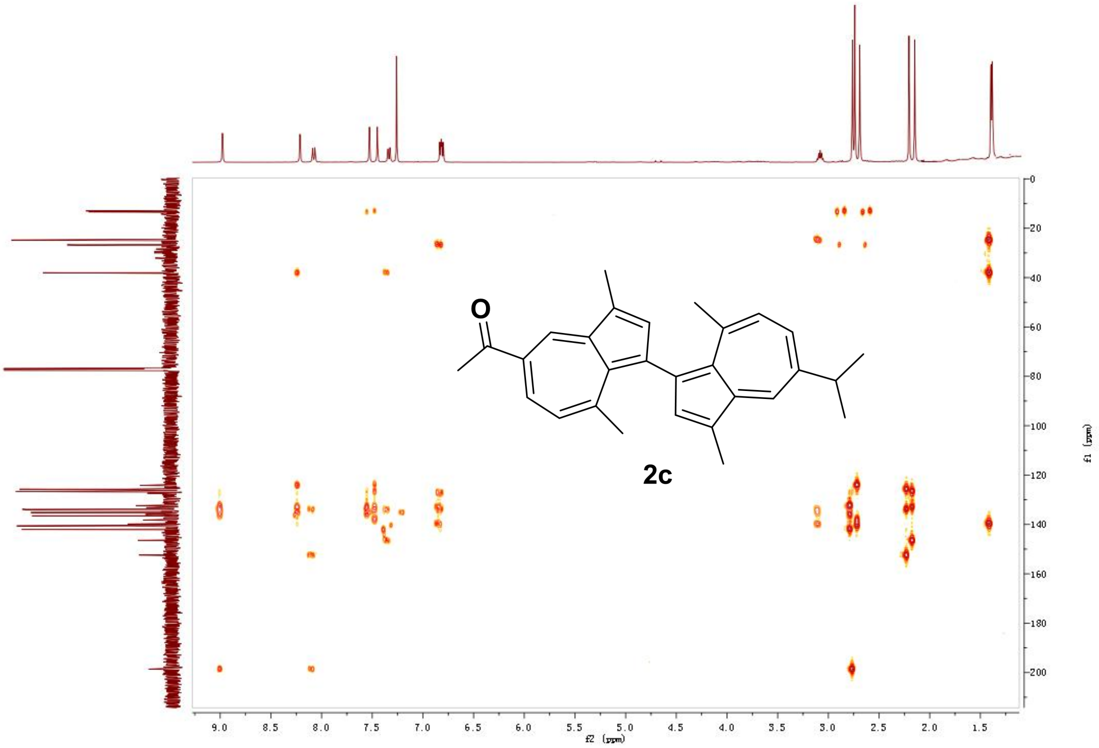

Figure S13. The HMBC spectrum of **2c** in $CDCl_3$ (500 MHz).

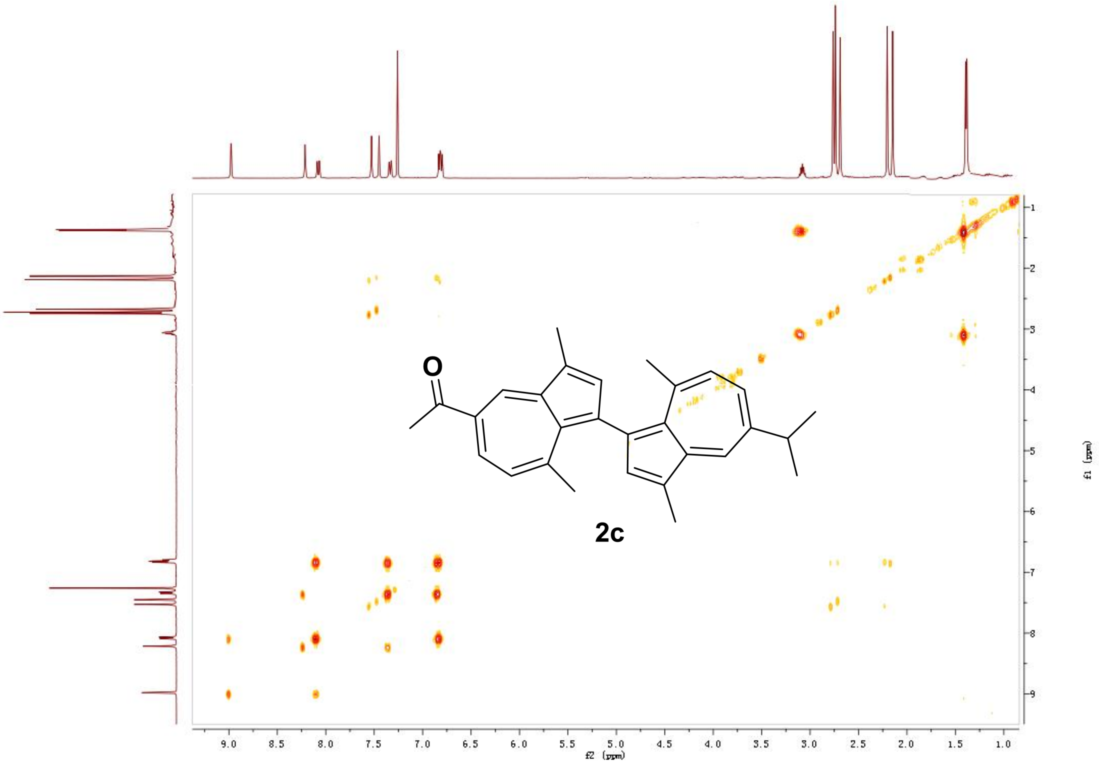

Figure S14. The ^1H-^1H COSY spectrum of **2c** in $CDCl_3$ (500 MHz).

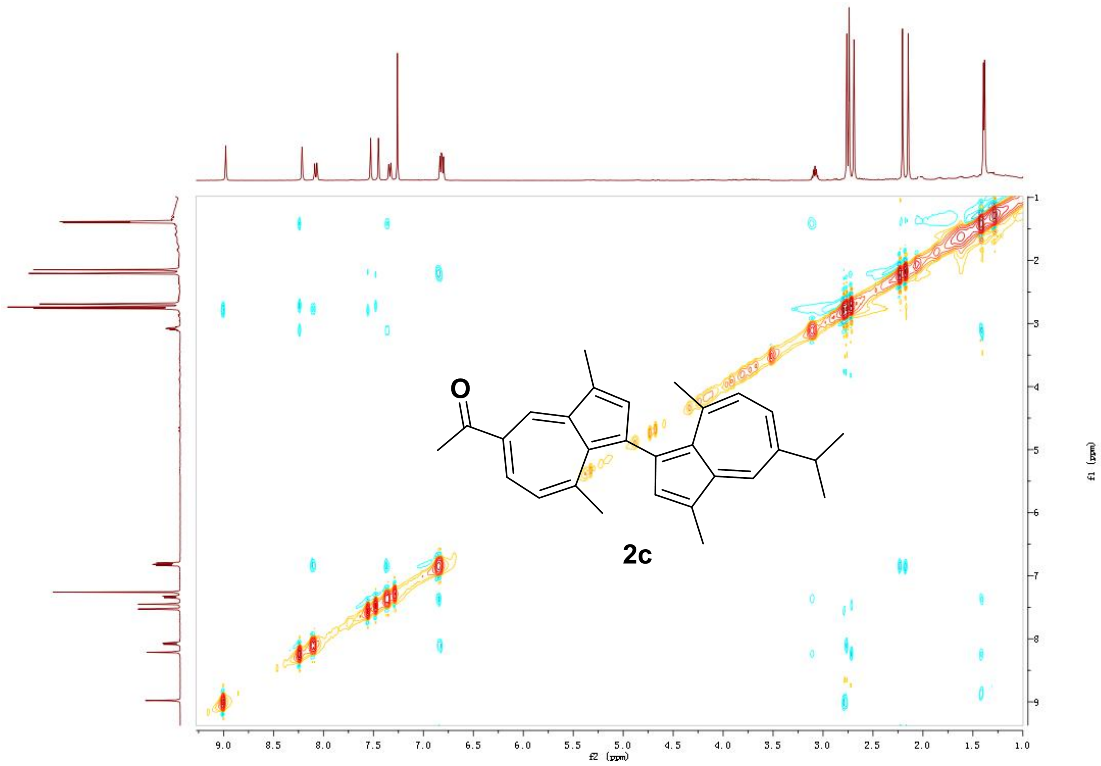

Figure S15. The NOESY spectrum of **2c** in $CDCl_3$ (500 MHz).

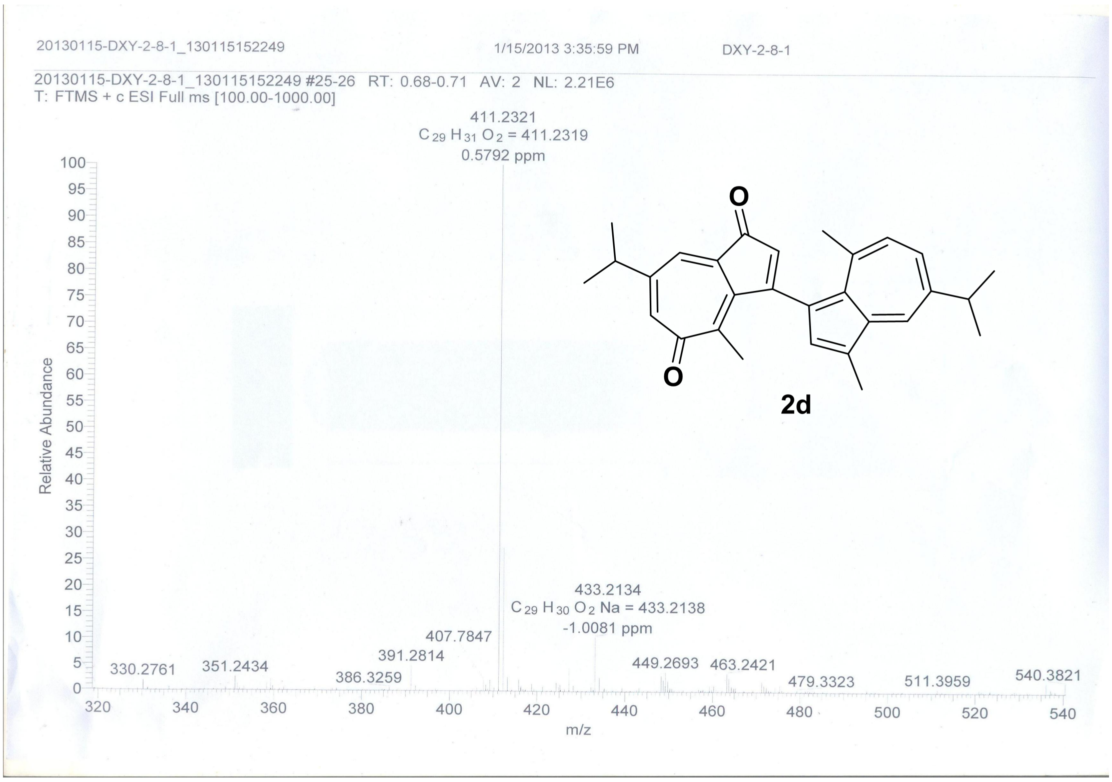

Figure S16. The HR-ESI-MS spectrum of **2d**.

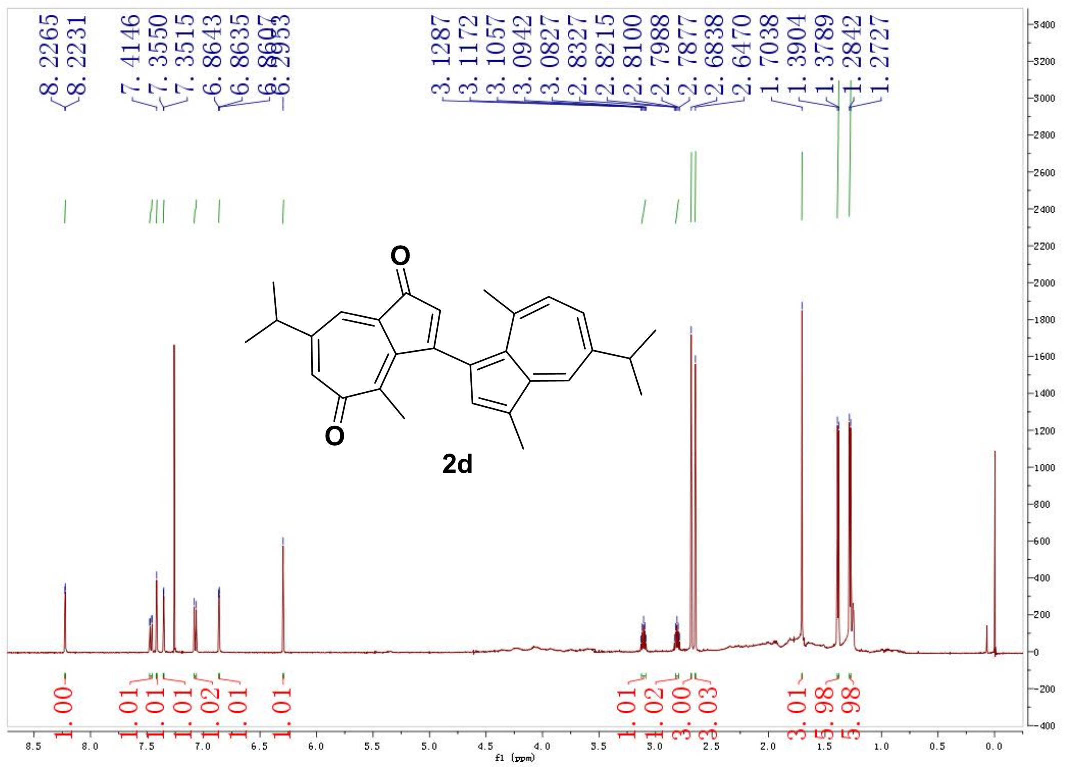

Figure S17. The ^{1}H-NMR spectrum of **2d** in $CDCl_3$ (600 MHz).

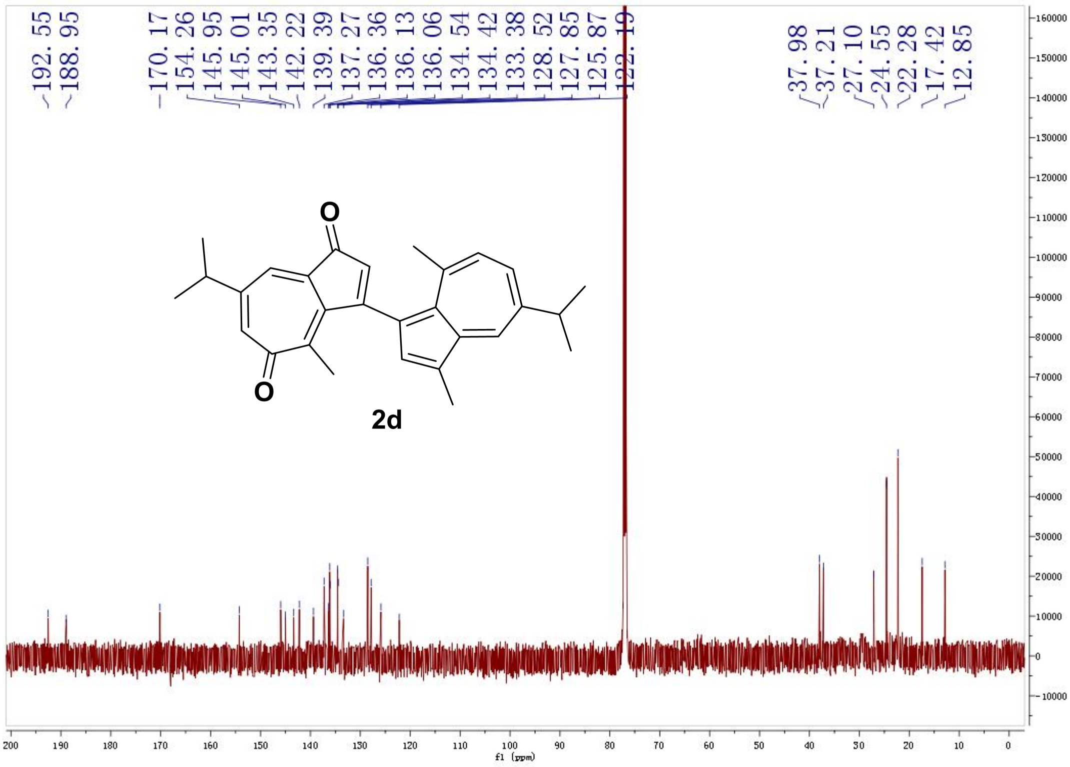

Figure S18. The ^{13}C-NMR spectrum of **2d** in $CDCl_3$ (150 MHz).

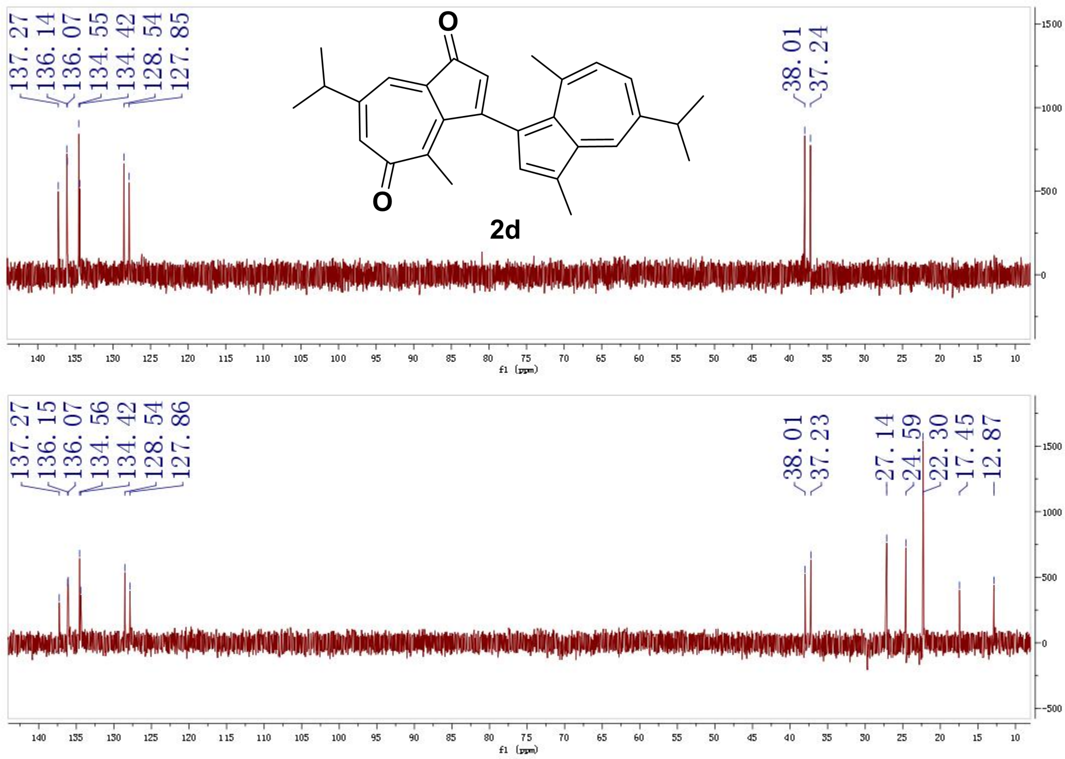

Figure S19. The DEPT spectrum of **2d** in $CDCl_3$ (150 MHz).

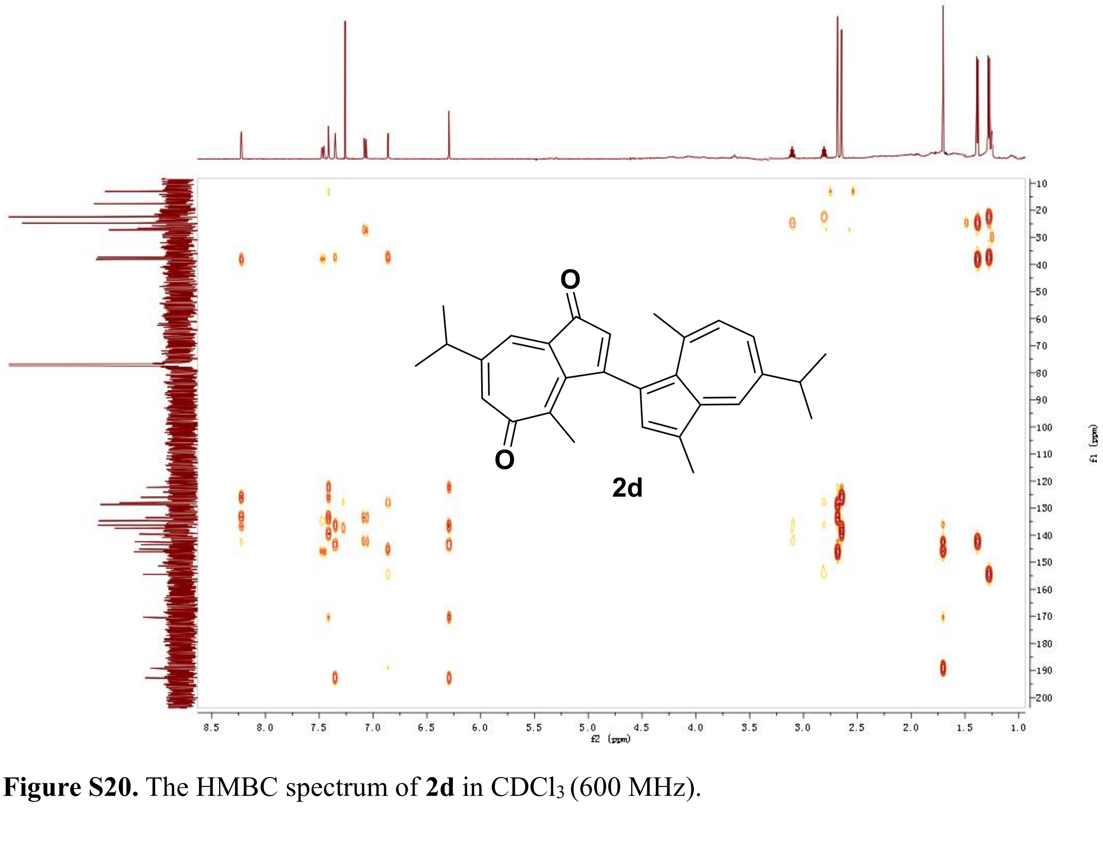

Figure S20. The HMBC spectrum of **2d** in $CDCl_3$ (600 MHz).

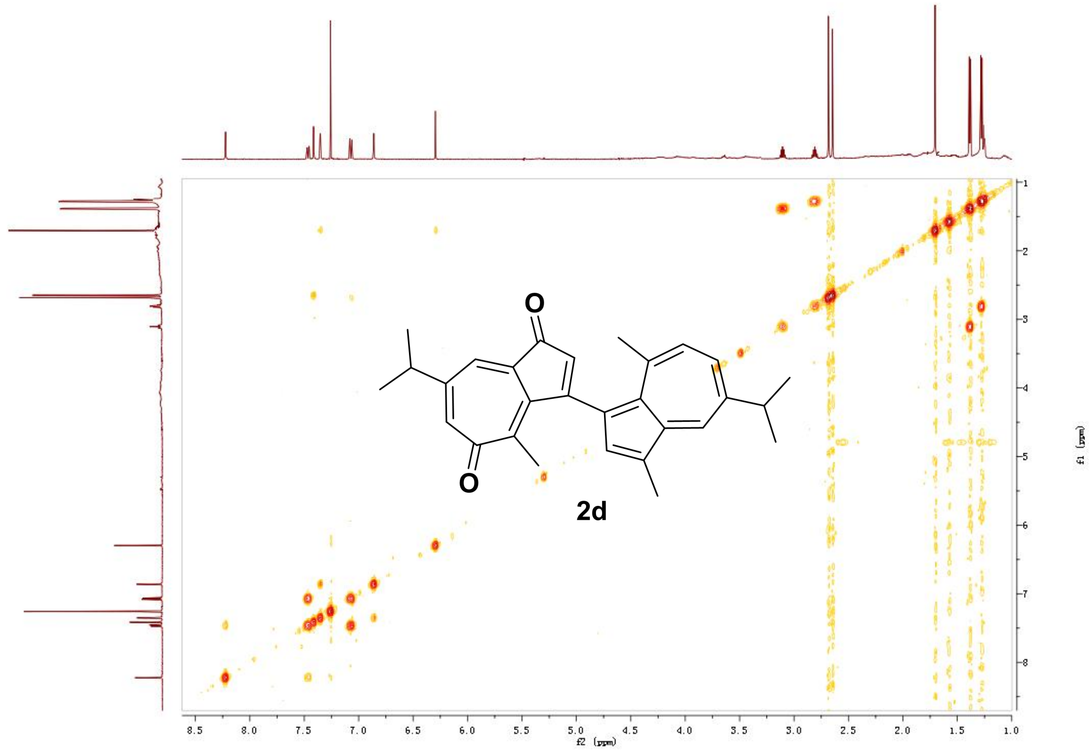

Figure S21. The ¹H-¹H COSY spectrum of **2d** in $CDCl_3$ (600 MHz).

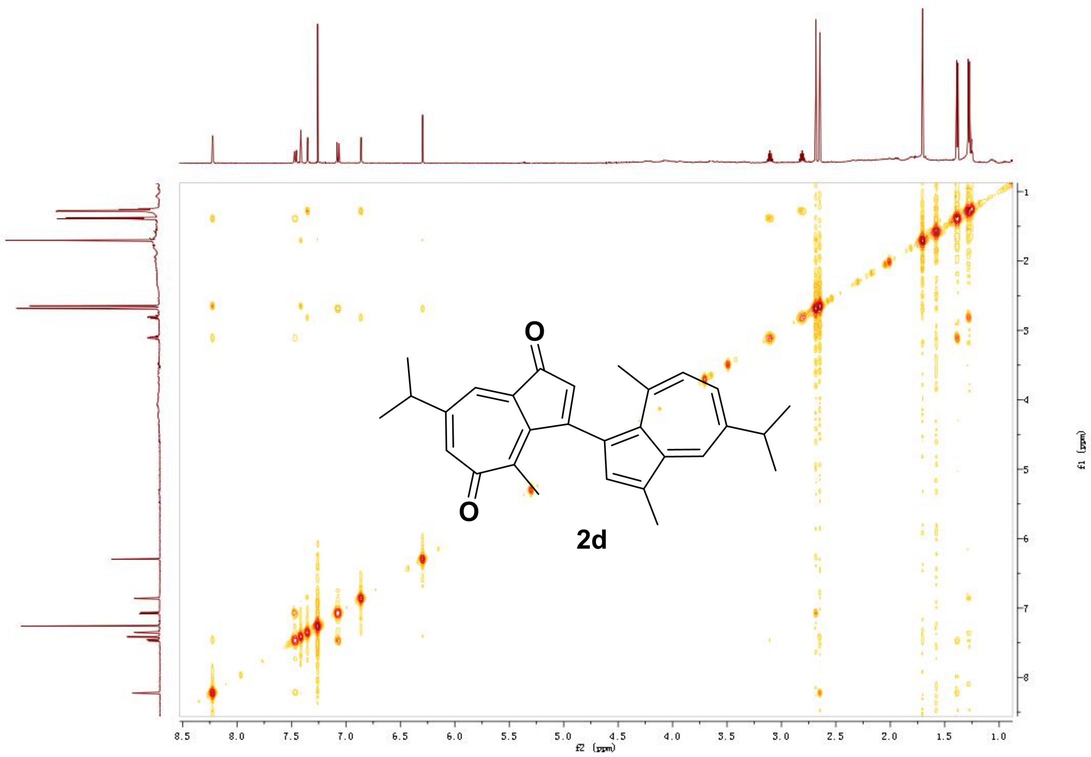

Figure S22. The NOESY spectrum of **2d** in $CDCl_3$ (600 MHz).

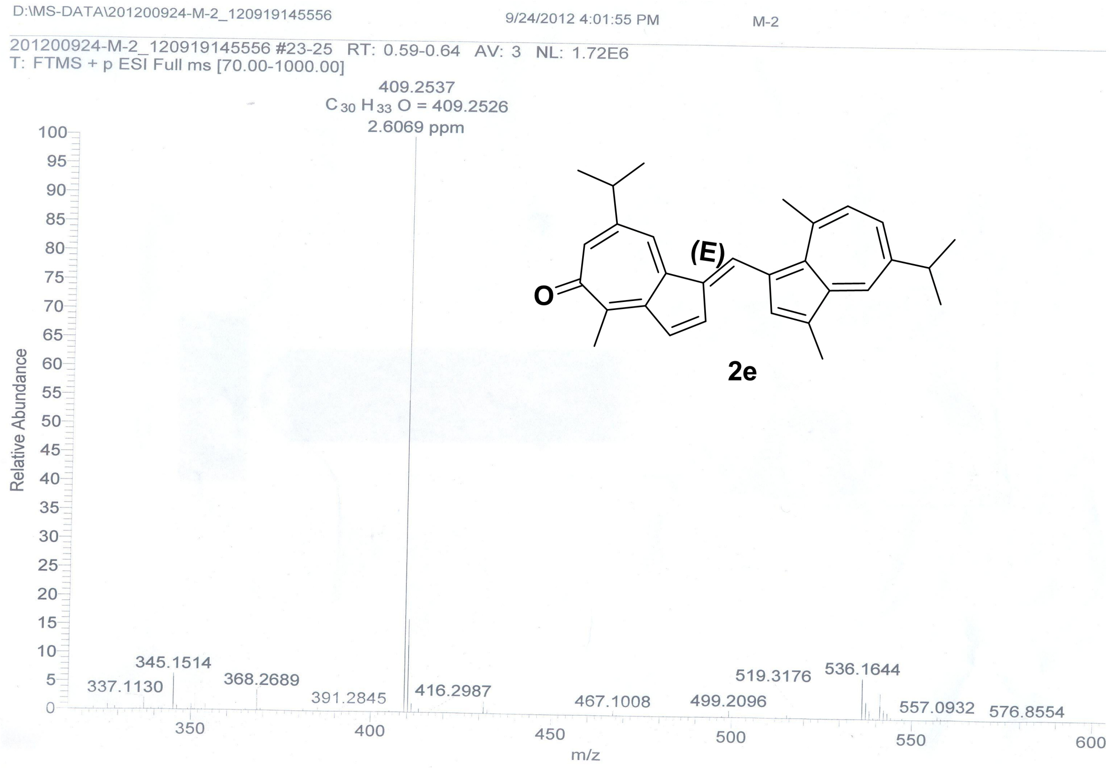

Figure S23. The HR-ESI-MS spectrum of **2e**.

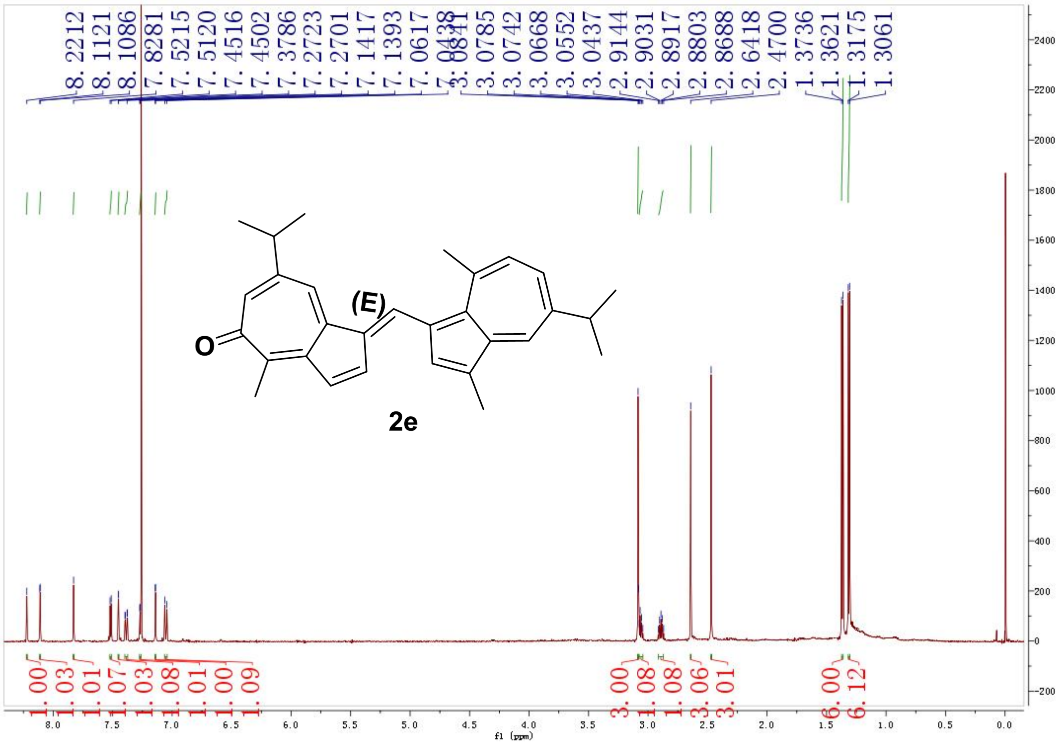

Figure S24. The ^{1}H-NMR spectrum of **2e** in $CDCl_3$ (600 MHz).

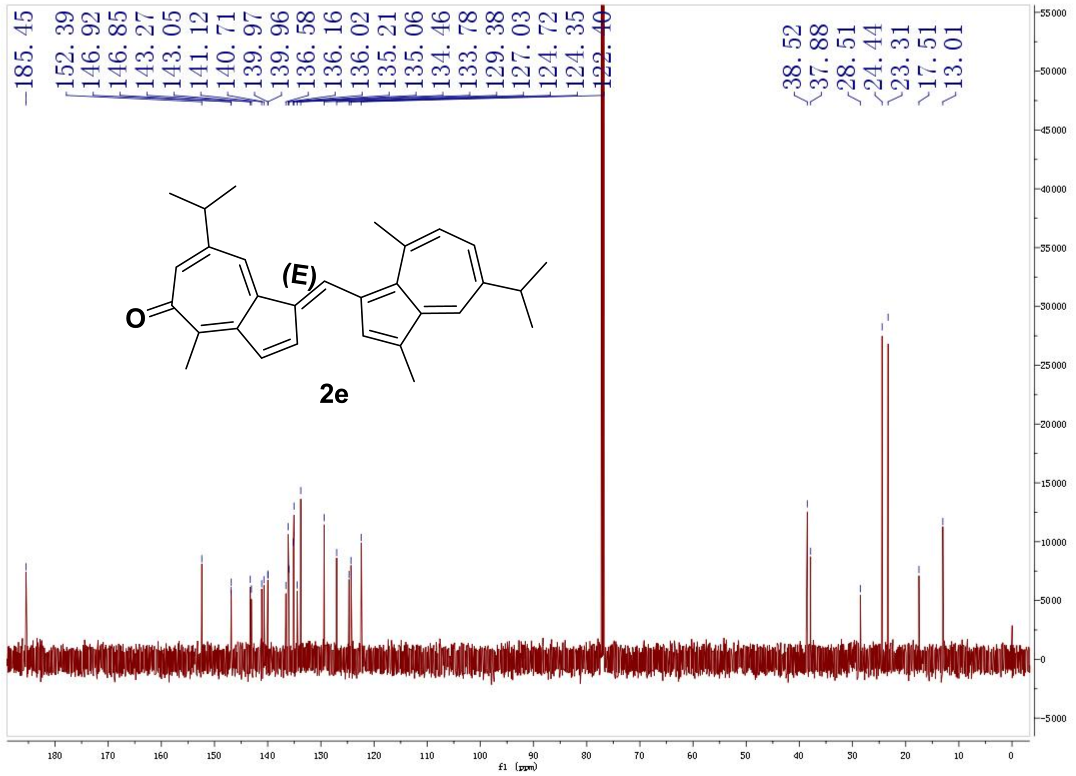

Figure S25. The ^{13}C-NMR spectrum of **2e** in $CDCl_3$ (150 MHz).

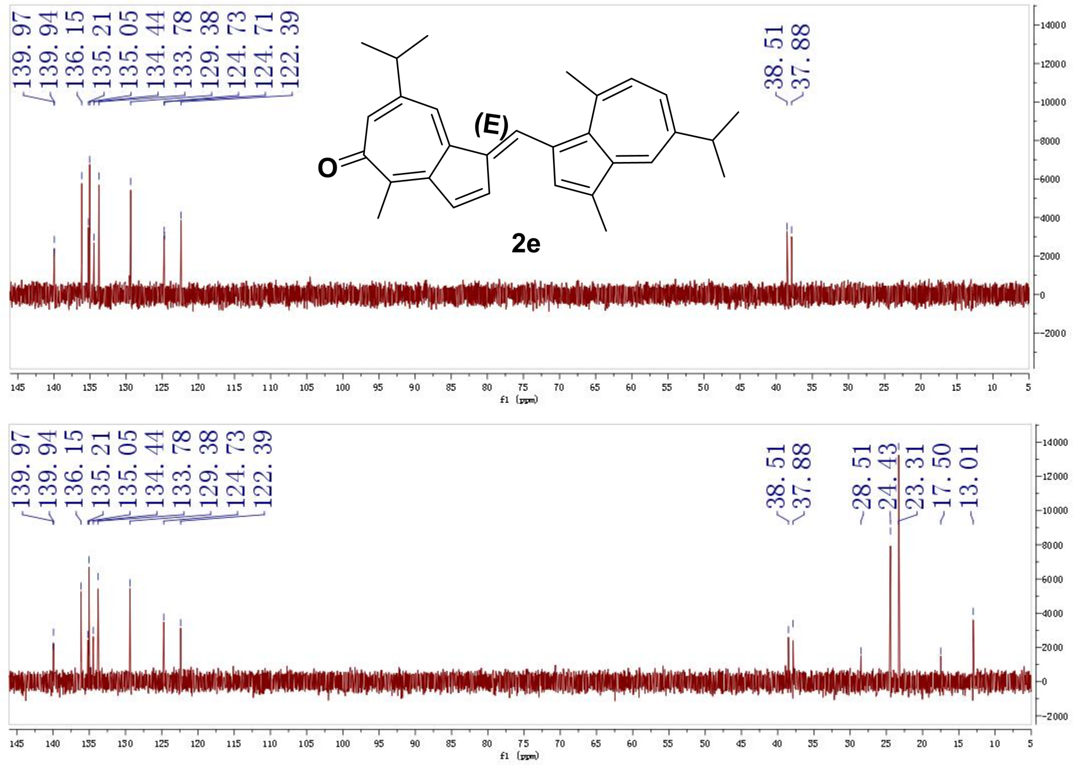

Figure S26. The DEPT spectrum of **2e** in $CDCl_3$ (150 MHz).

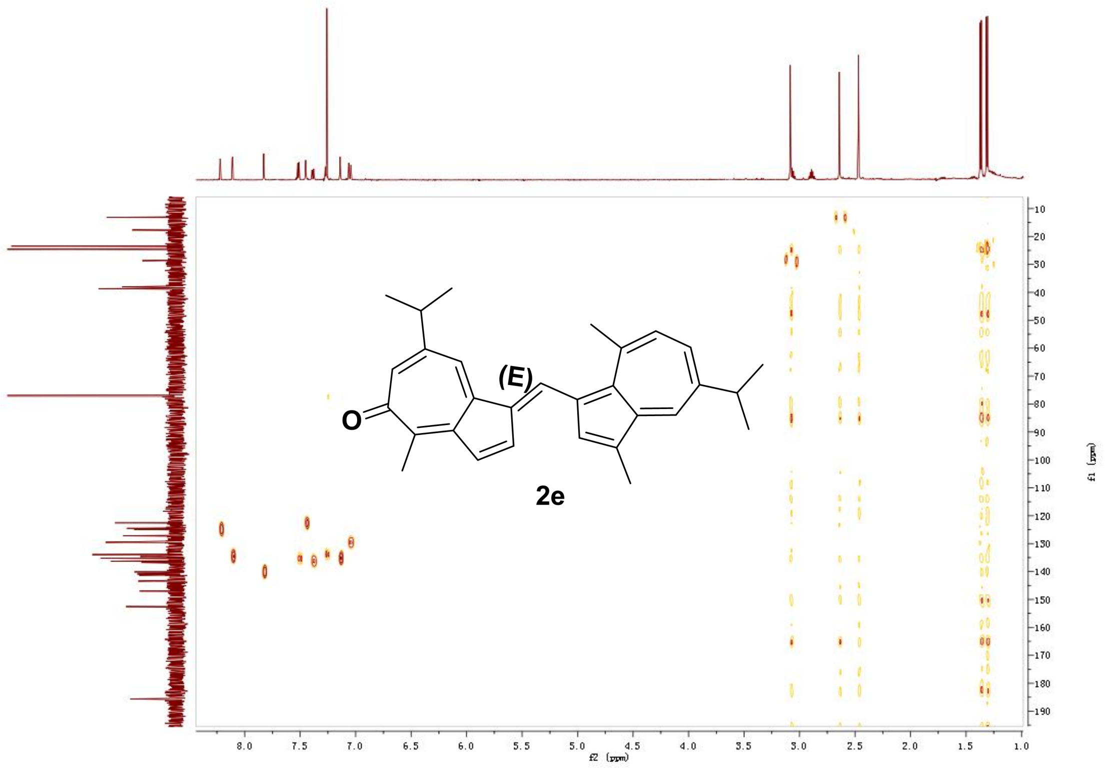

Figure S27. The HMQC spectrum of **2e** in $CDCl_3$ (600 MHz).

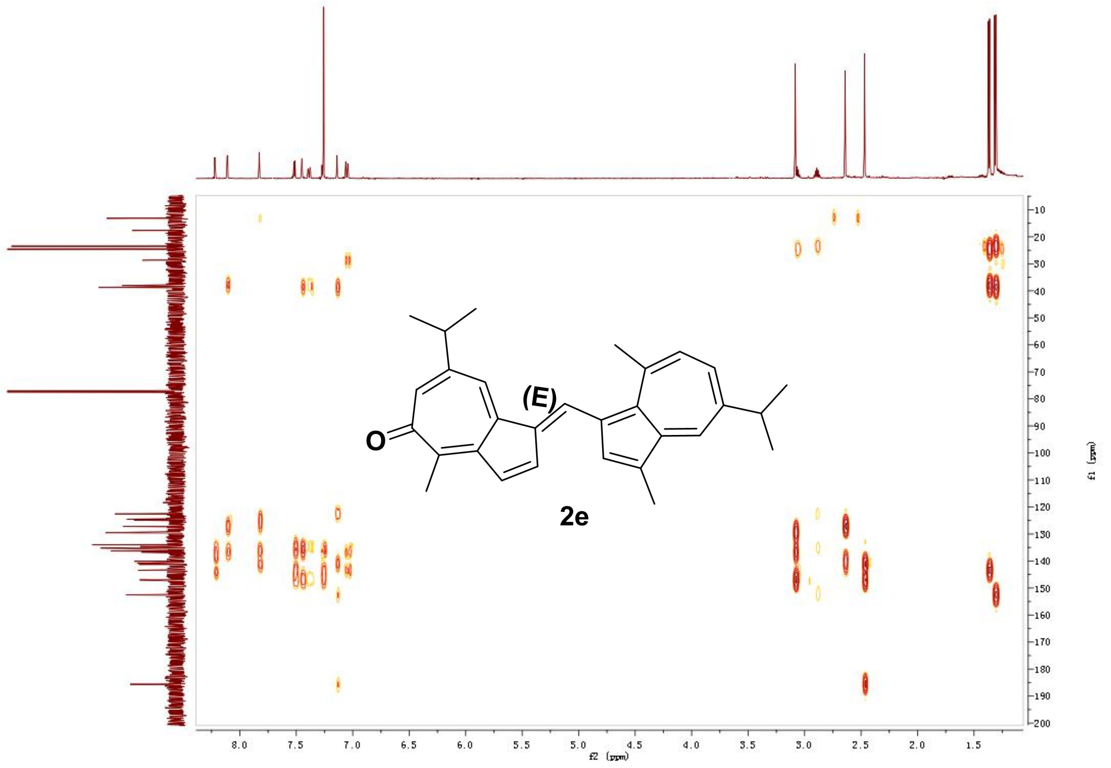

Figure S28. The HMBC spectrum of **2e** in $CDCl_3$ (600 MHz).

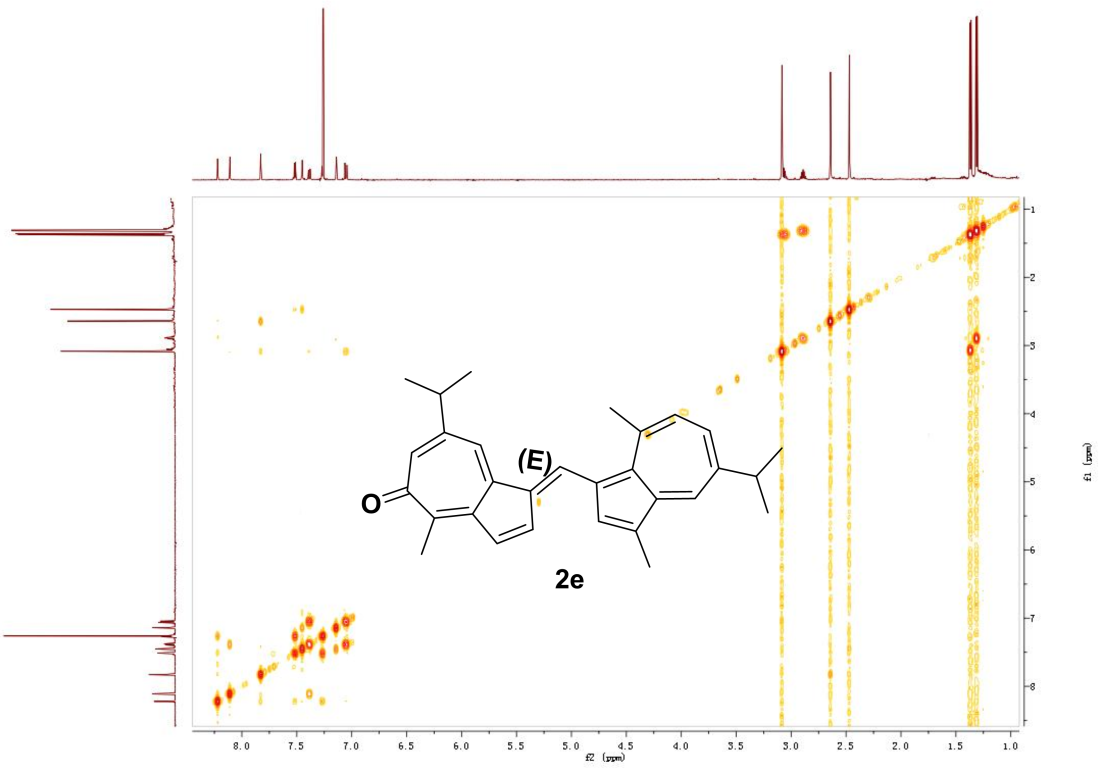

Figure S29. The ^{1}H-^{1}H COSY spectrum of **2e** in $CDCl_3$ (600 MHz).

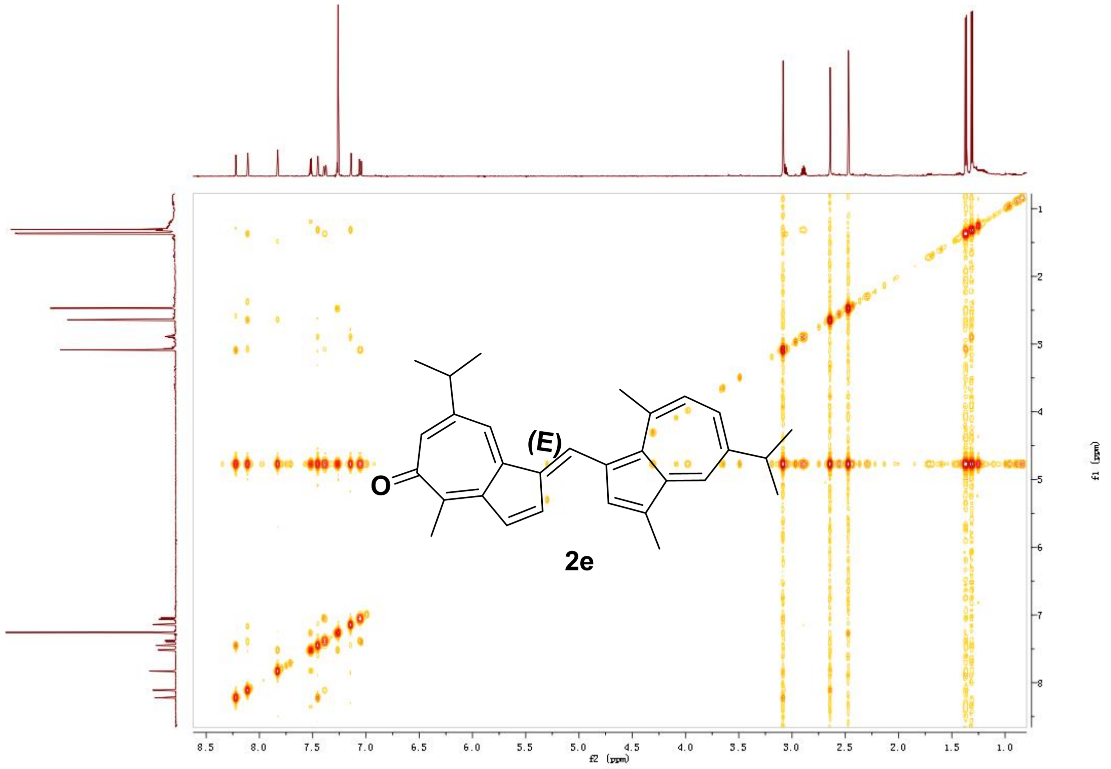

Figure S30. The NOESY spectrum of **2e** in $CDCl_3$ (600 MHz).

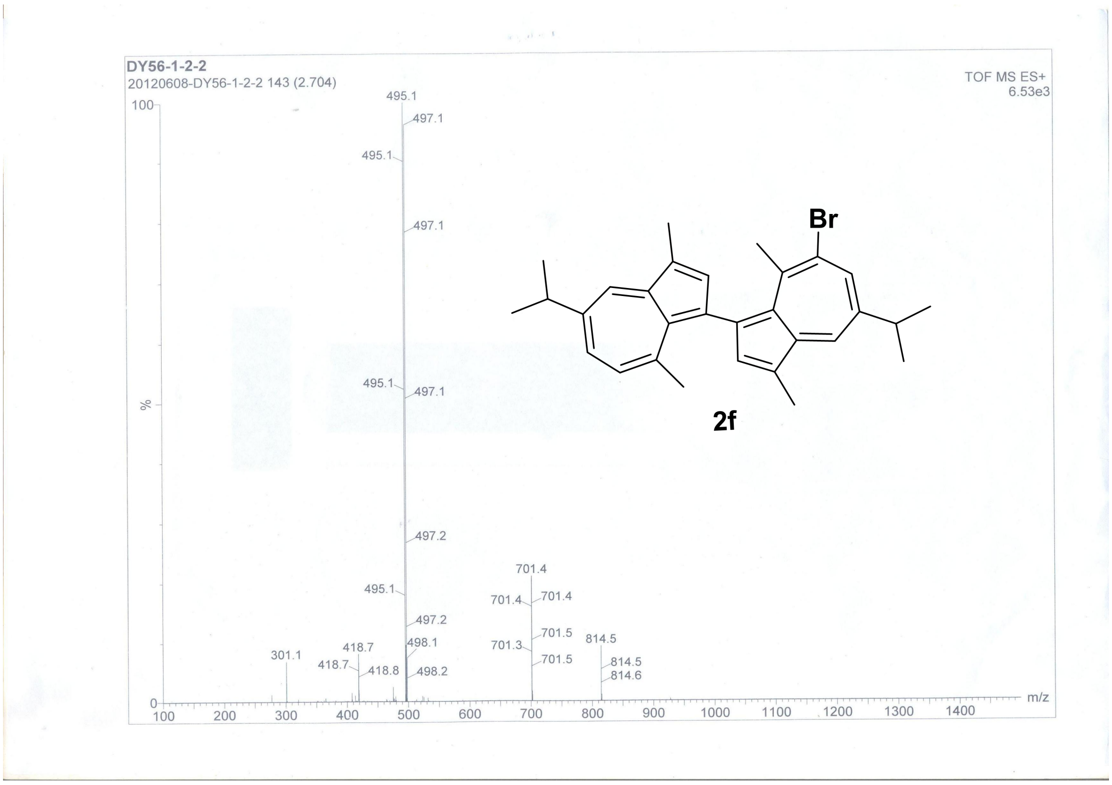

Figure S31. The ESI-MS spectrum of **2f**.

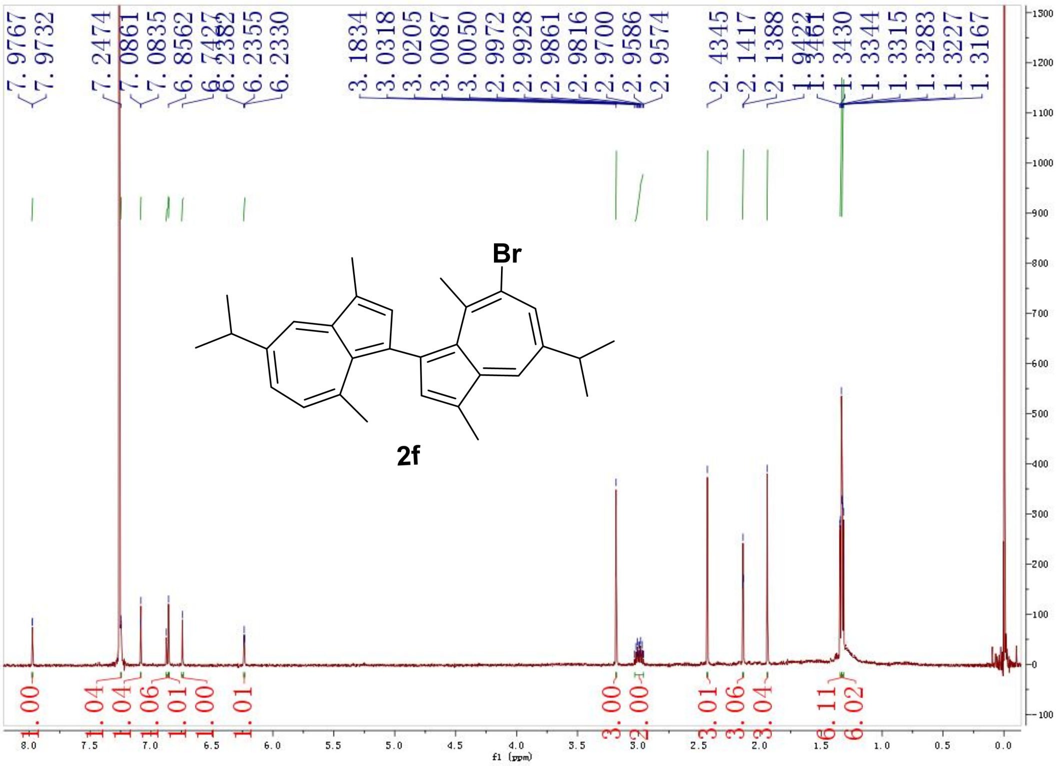

Figure S32. The ^{1}H-NMR spectrum of **2f** in $CDCl_3$ (600 MHz).

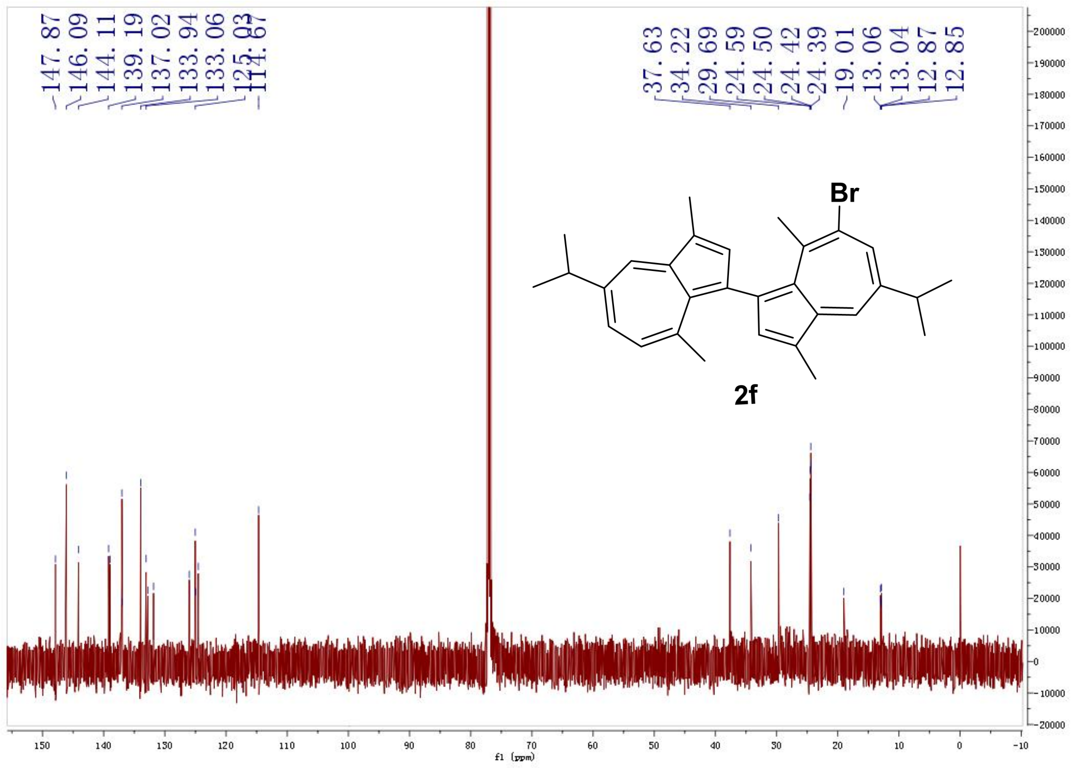

Figure S33. The ^{13}C-NMR spectrum of **2f** in $CDCl_3$ (150 MHz).

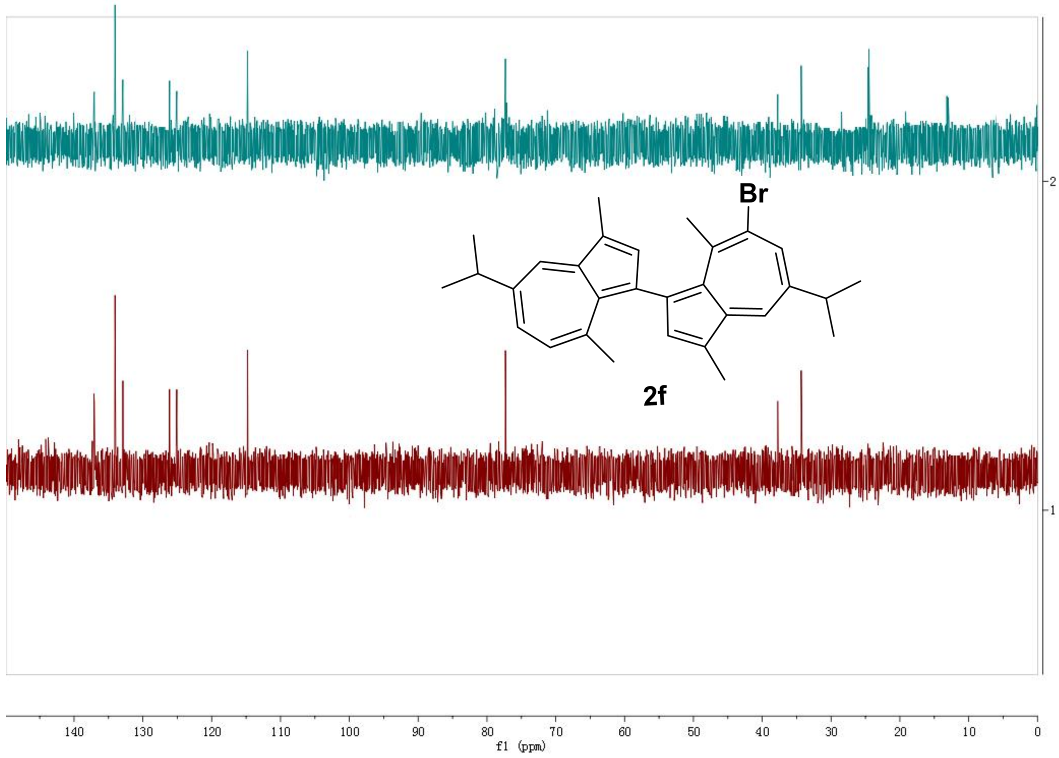

Figure S34. The DEPT spectrum of **2f** in $CDCl_3$ (150 MHz).

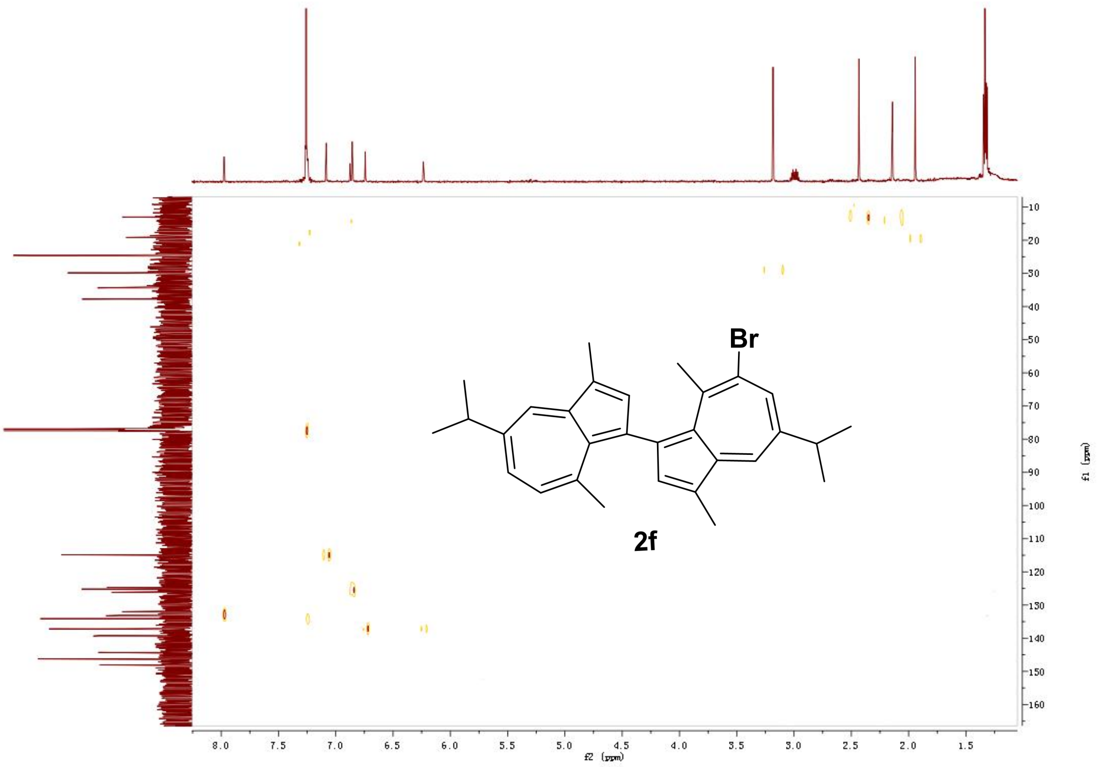

Figure S35. The HMQC spectrum of **2f** in $CDCl_3$ (600 MHz).

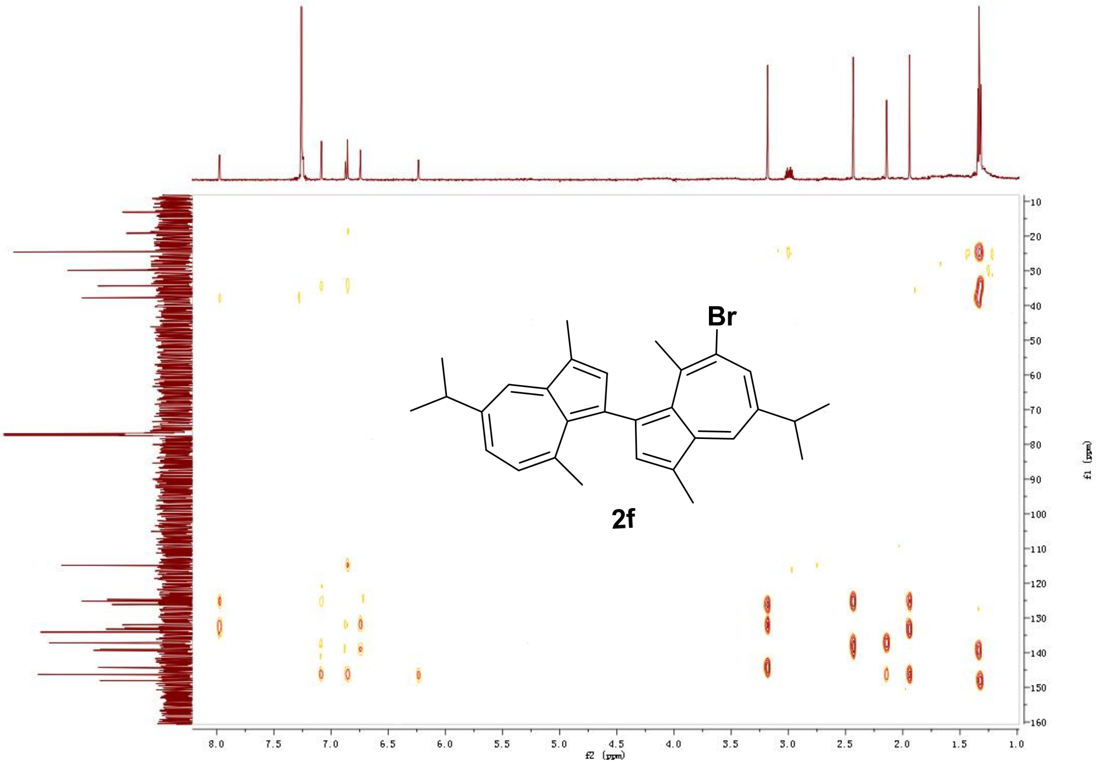

Figure S36. The HMBC spectrum of **2f** in $CDCl_3$ (600 MHz).

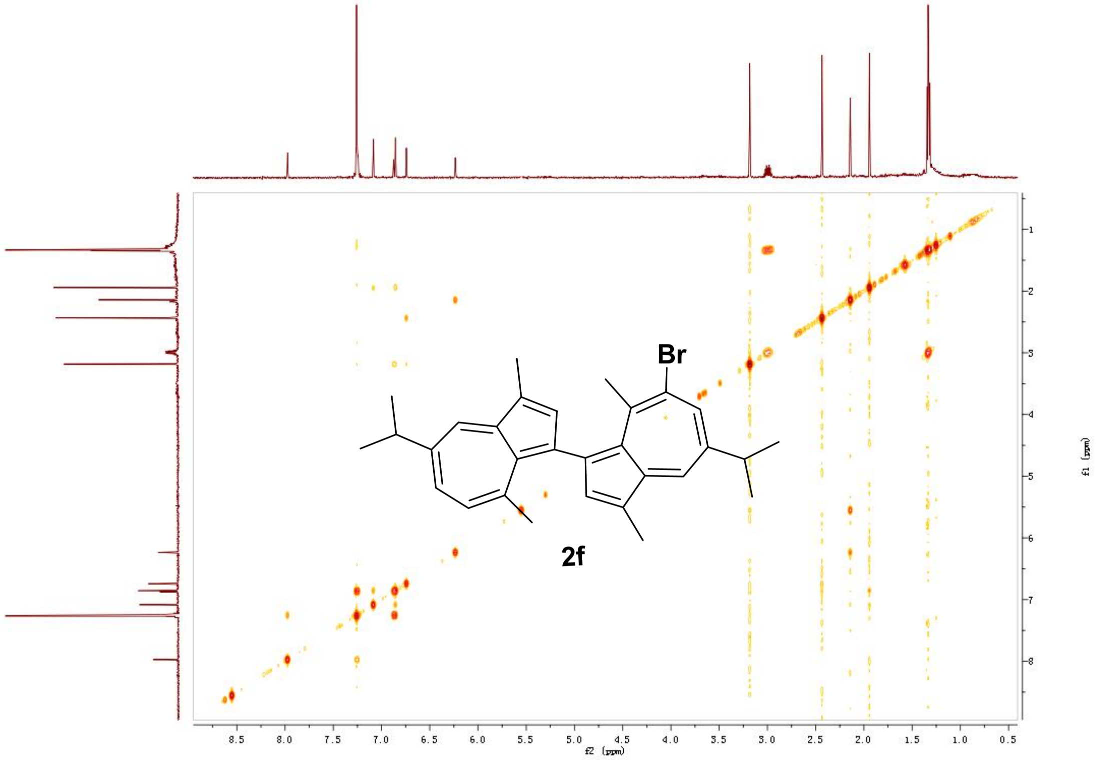

Figure S37. The ^{1}H-^{1}H COSY spectrum of **2f** in $CDCl_3$ (600 MHz).

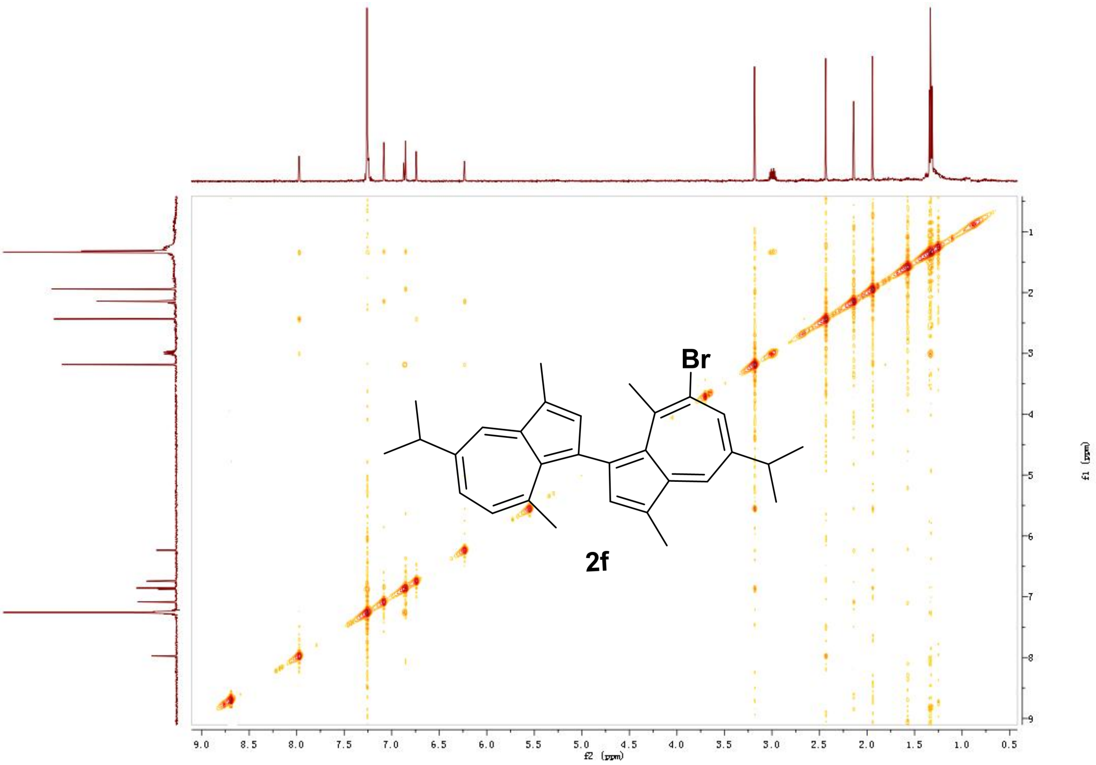

Figure S38. The NOESY spectrum of **2f** in $CDCl_3$ (600 MHz).

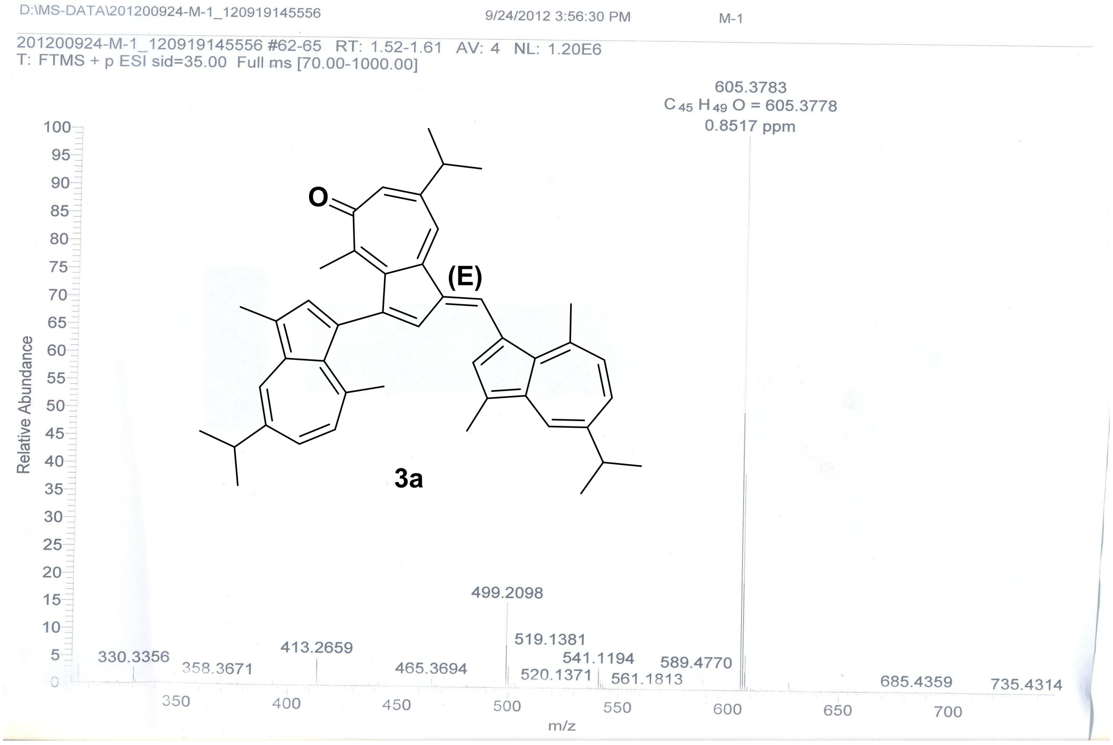

Figure S39. The HR-ESI-MS spectrum of **3a**.

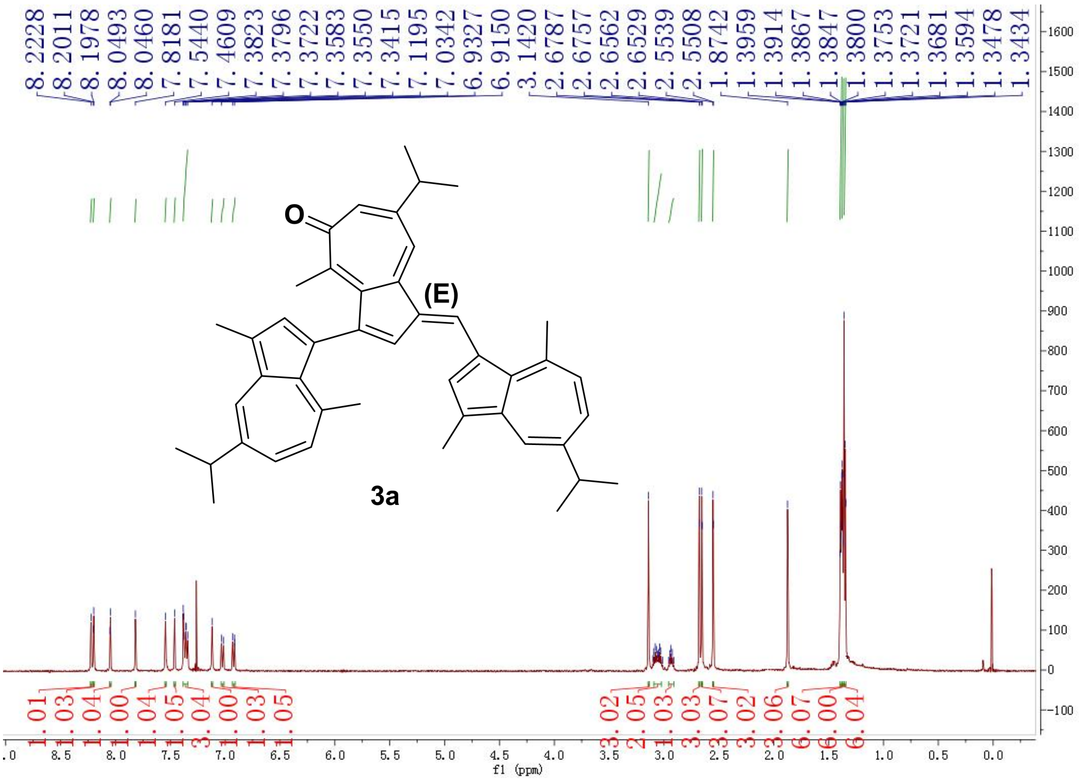

Figure S40. The ^{1}H-NMR spectrum of **3a** in $CDCl_3$ (600 MHz).

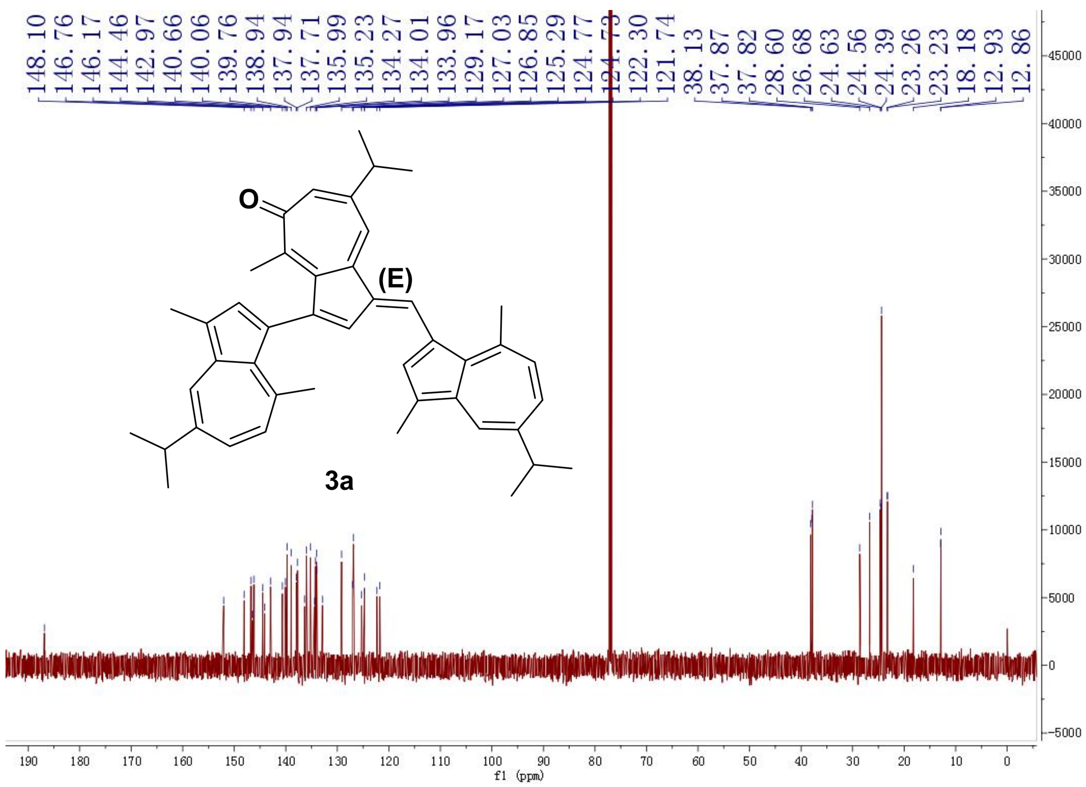

Figure S41. The ^{13}C-NMR spectrum of **3a** in $CDCl_3$ (150 MHz).

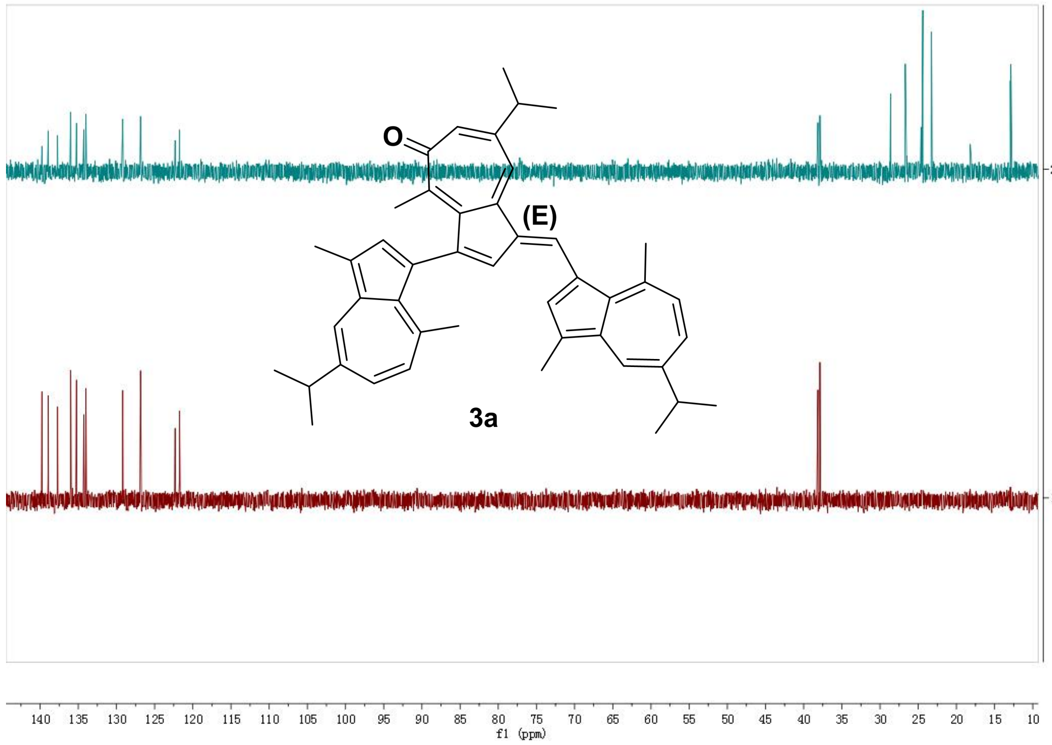

Figure S42. The DEPT spectrum of **3a** in $CDCl_3$ (150 MHz).

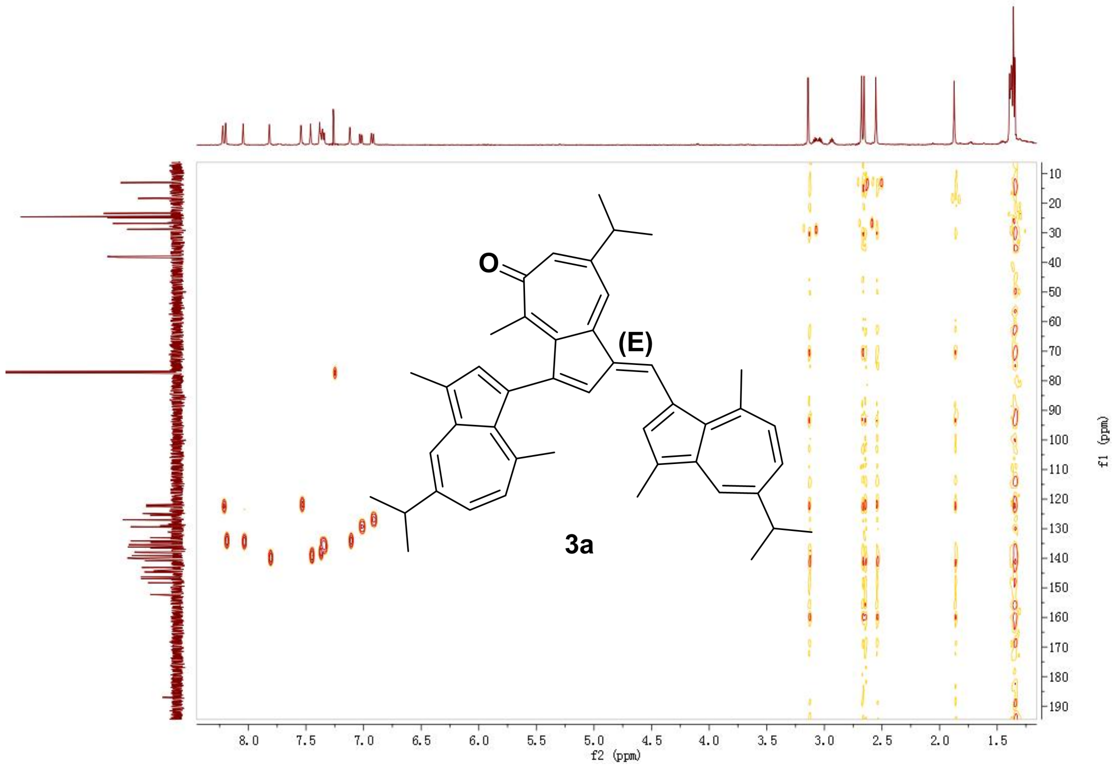

Figure S43. The HMQC spectrum of **3a** in $CDCl_3$ (600 MHz).

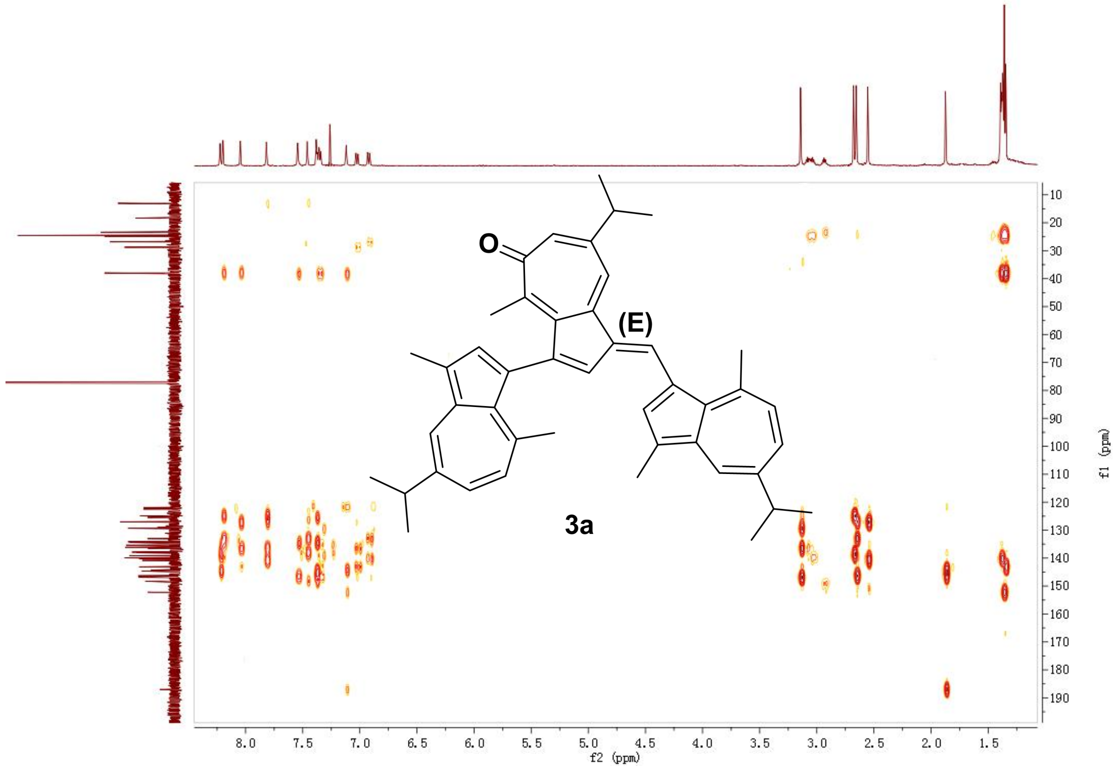

Figure S44. The HMBC spectrum of **3a** in $CDCl_3$ (600 MHz).

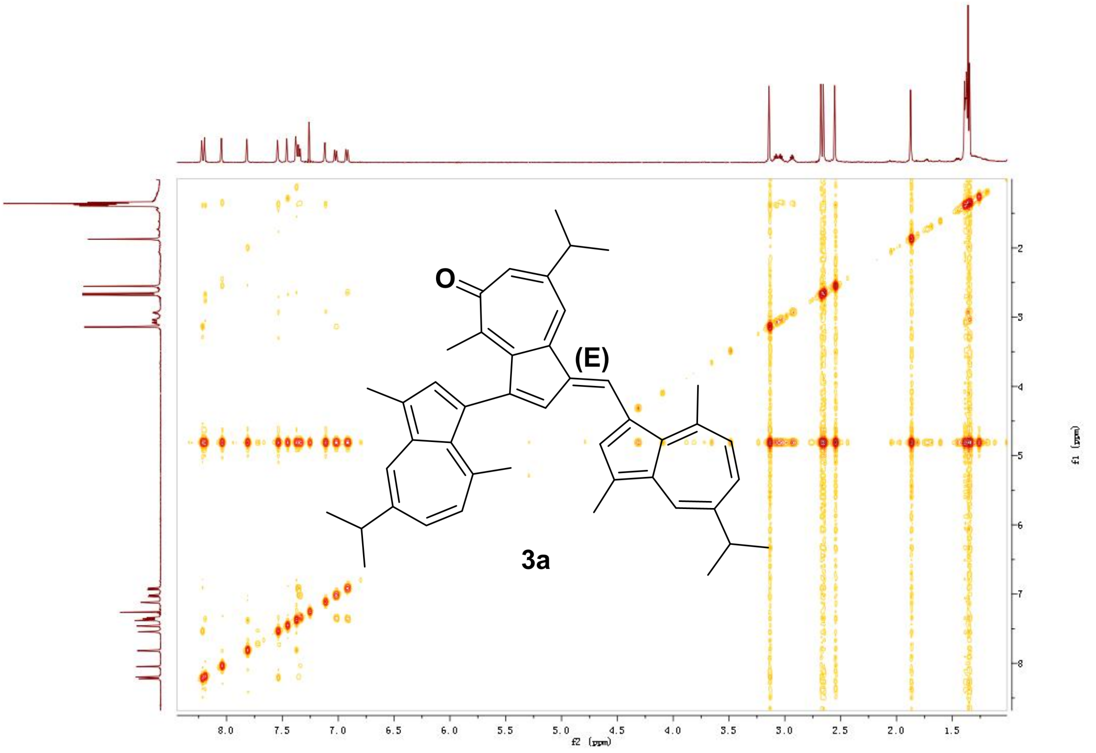

Figure S45. The NOESY spectrum of **3a** in $CDCl_3$ (600 MHz).

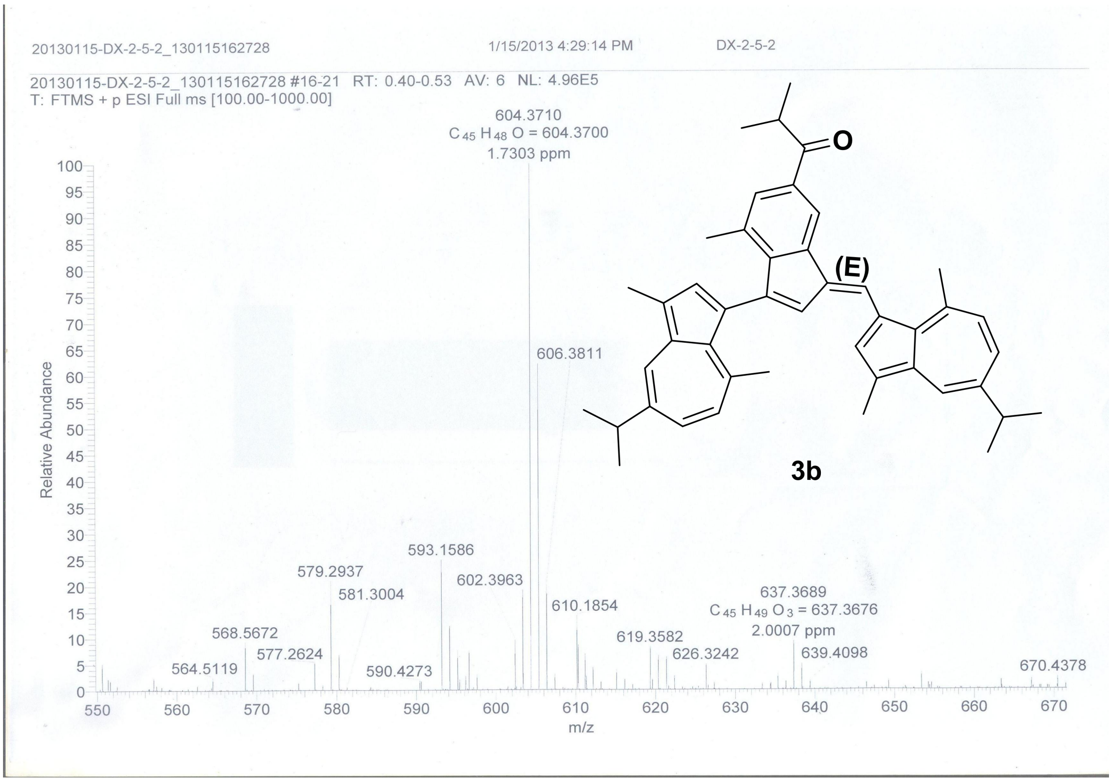

Figure S46. The HR-ESI-MS spectrum of **3b**.

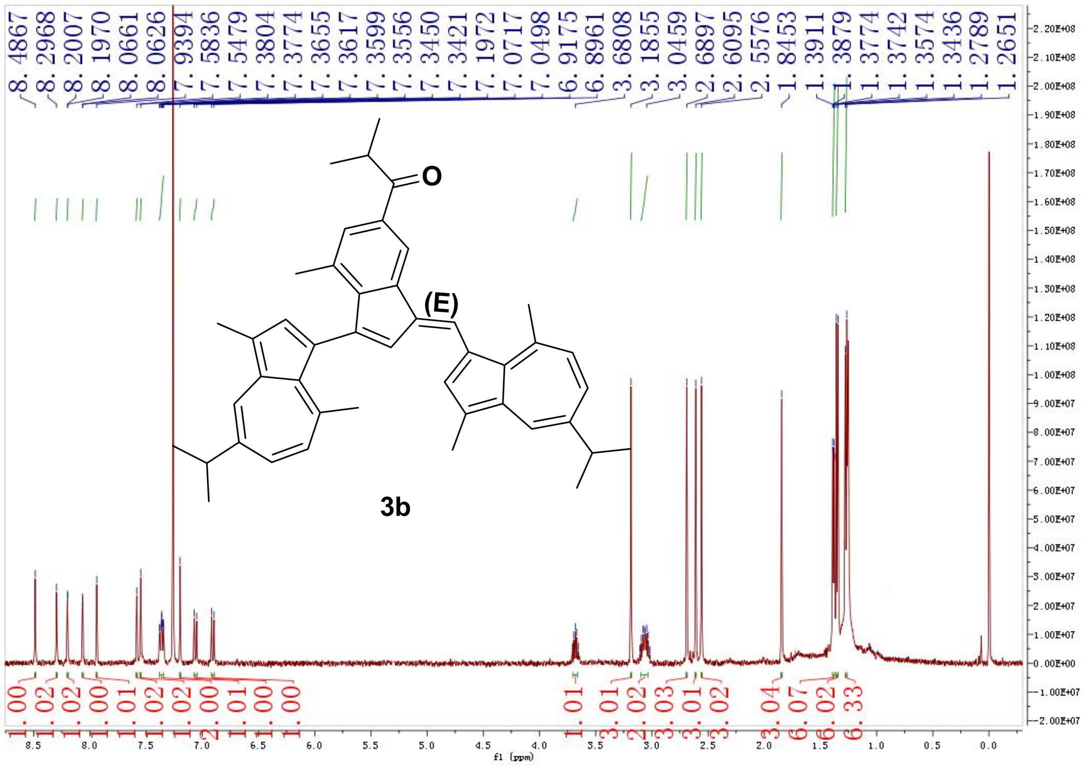

Figure S47. The ^{1}H-NMR spectrum of **3b** in $CDCl_3$ (500 MHz).

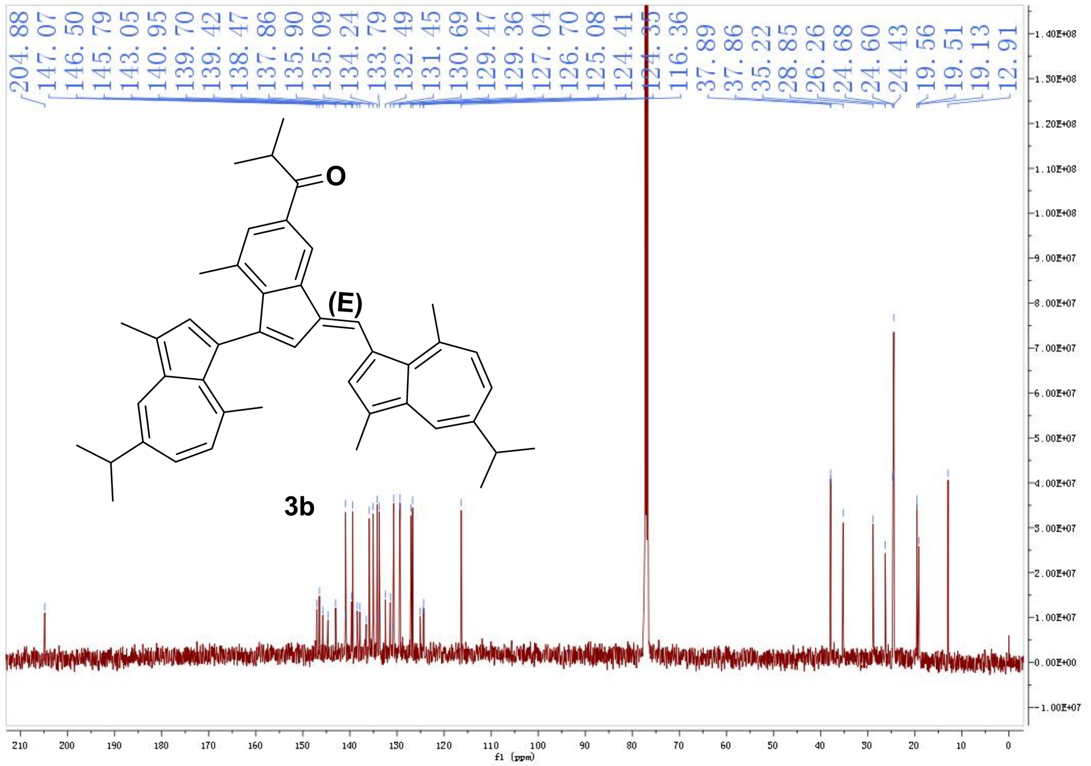

Figure S48. The ^{13}C-NMR spectrum of **3b** in $CDCl_3$ (125 MHz).

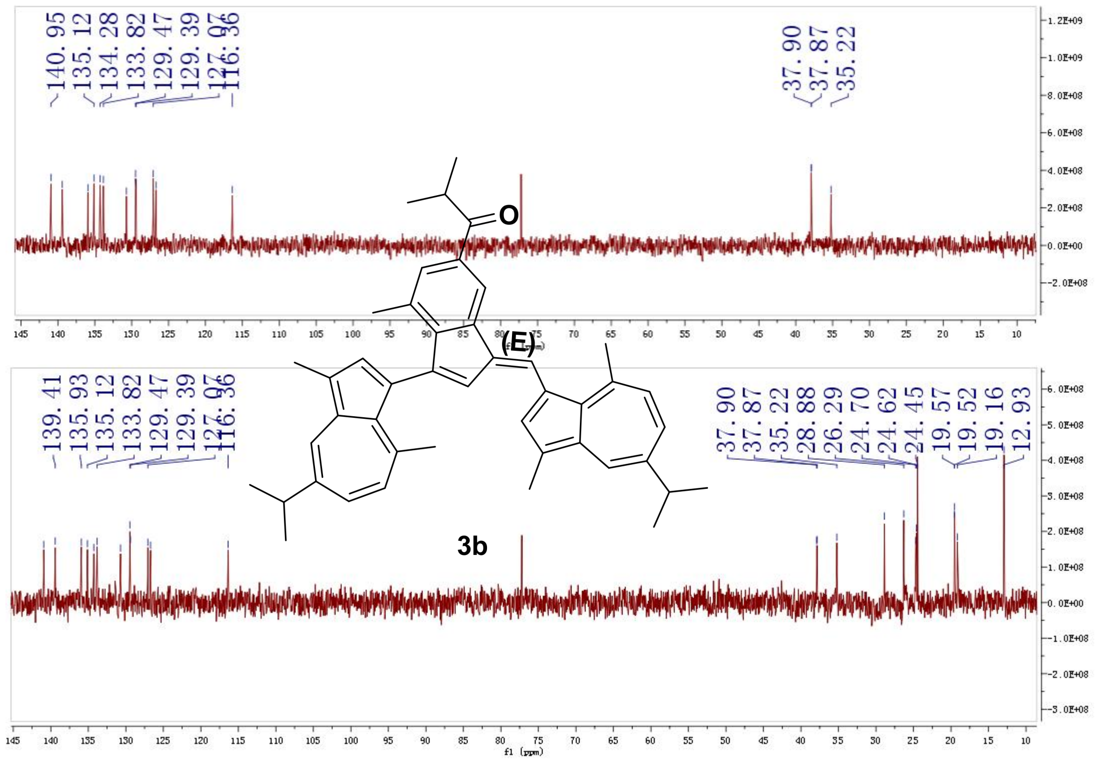

Figure S49. The DEPT spectrum of **3b** in $CDCl_3$ (125 MHz).

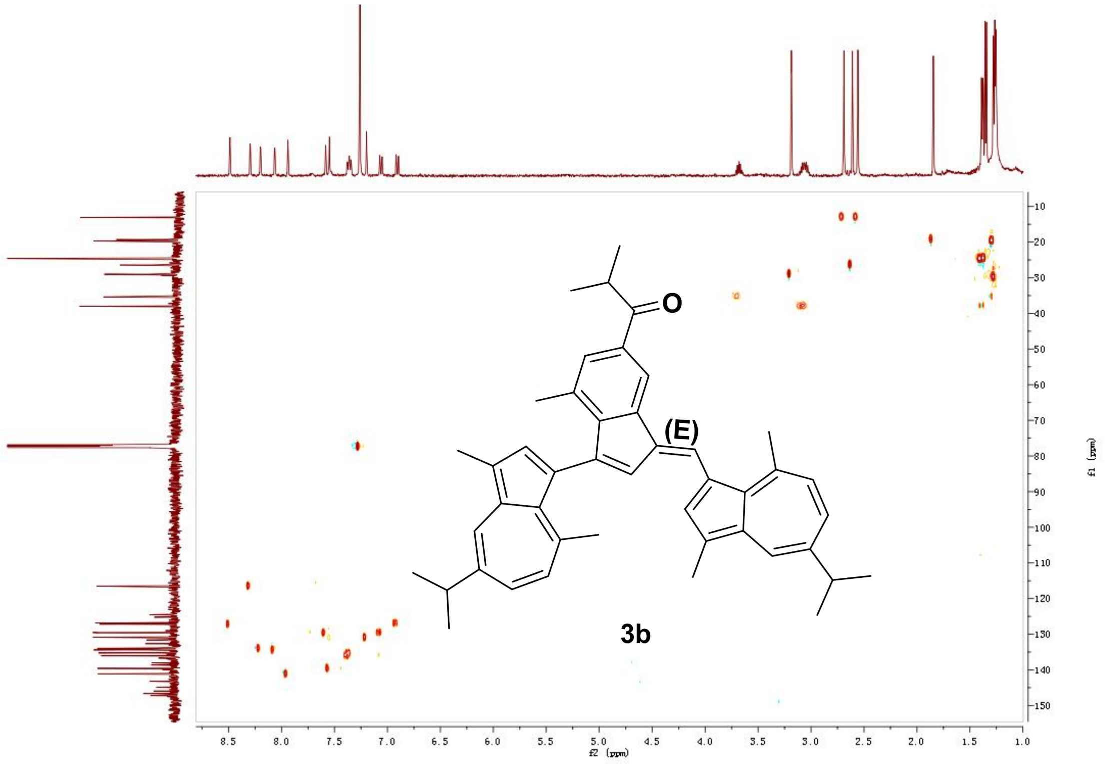

Figure S50. The HMQC spectrum of **3b** in $CDCl_3$ (500 MHz).

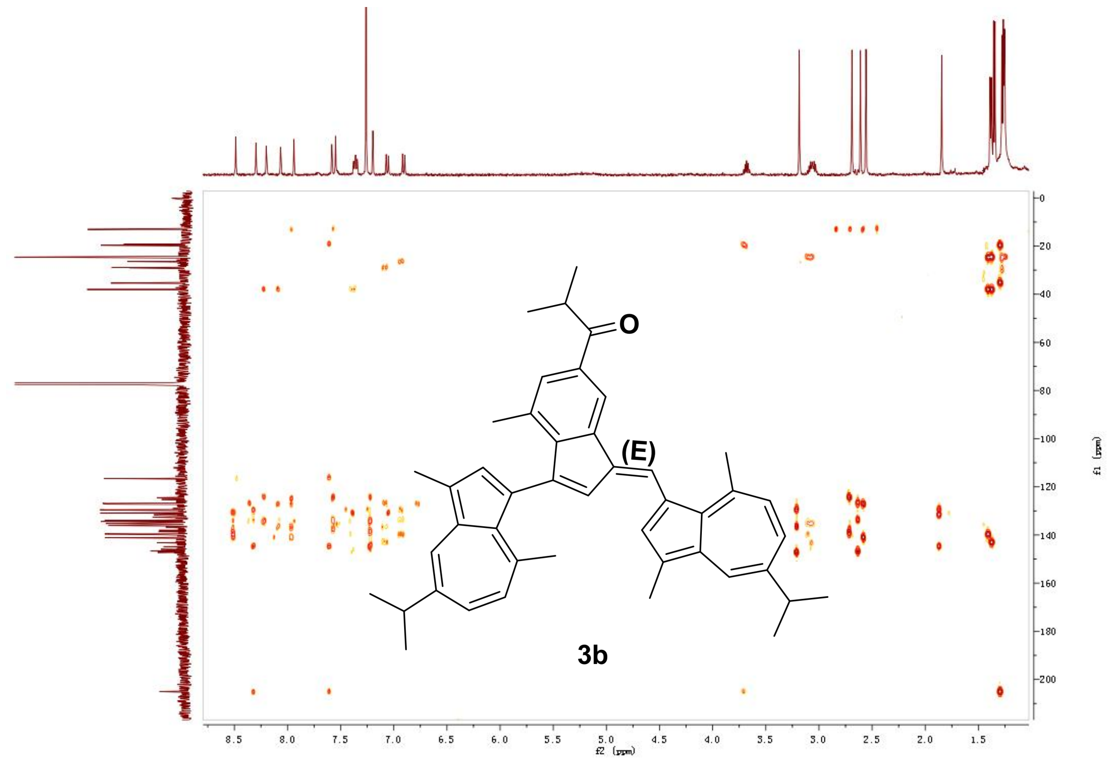

Figure S51. The HMBC spectrum of **3b** in $CDCl_3$ (500 MHz).

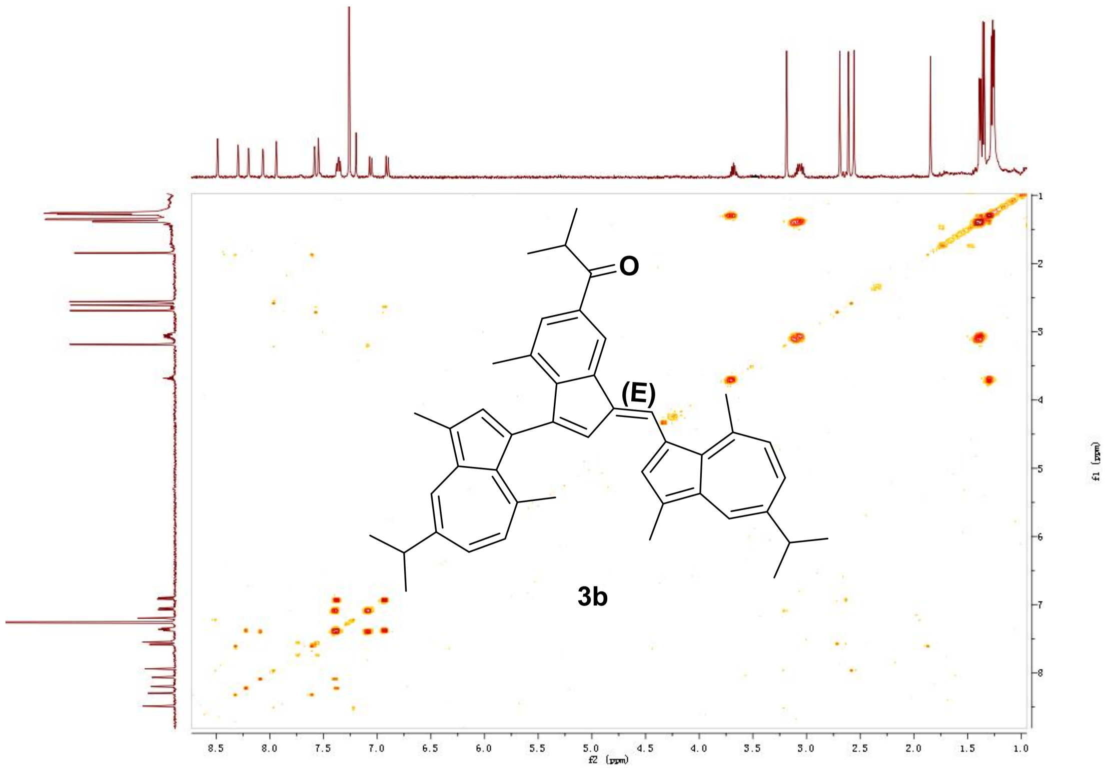

Figure S52. The ^1H-^1H COSY spectrum of **3b** in $CDCl_3$ (500 MHz).

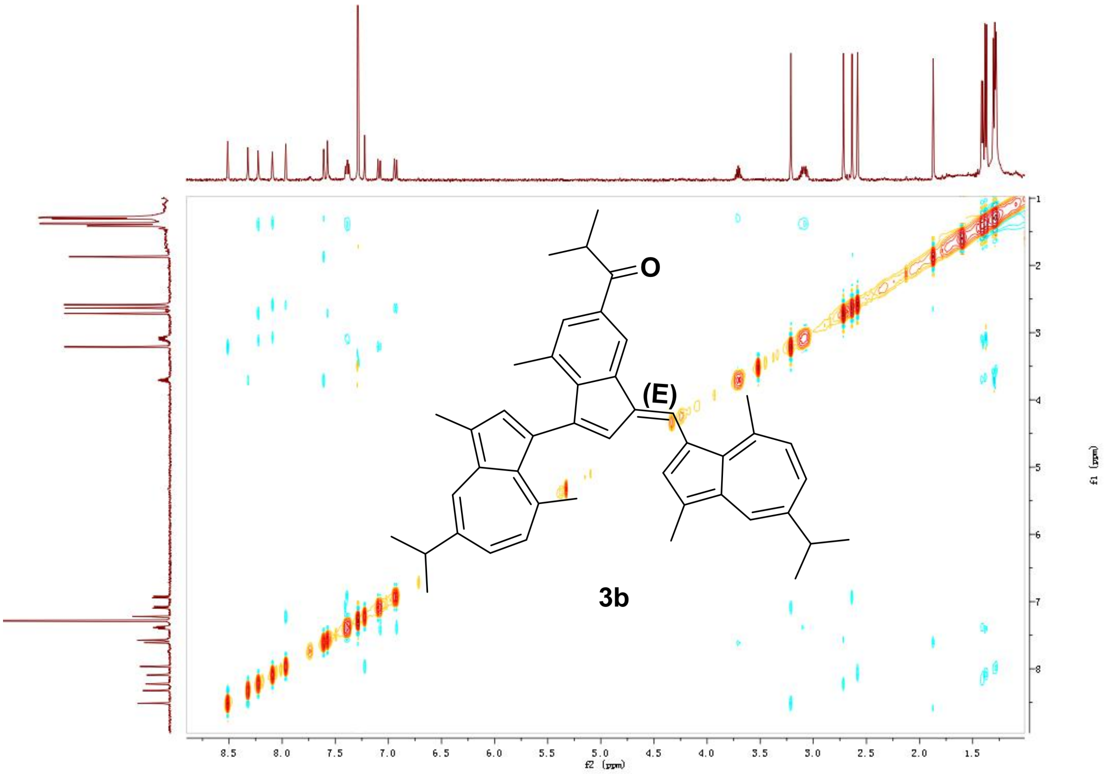

Figure S53. The NOESY spectrum of **3b** in $CDCl_3$ (500 MHz).
